# Unraveling the Crystal Structures of Picolinic Acid Derivatives: Synthesis, Packing, Interactions, and Conformational Flexibility

**DOI:** 10.1002/open.202500197

**Published:** 2025-05-02

**Authors:** Sara Camorali, Lorenzo Tei, Marco Milanesio, Mattia Lopresti

**Affiliations:** ^1^ Dipartimento di Scienze e Innovazione Tecnologica Università del Piemonte Orientale Viale Teresa Michel 11 15121 Alessandria Italy

**Keywords:** conformational modeling, Hirshfeld surface analysis, picolinic acid derivatives, structure determination, X‐ray diffraction

## Abstract

Picolinic acid and its derivatives are widely used as pendant arms in ligands for metal ion complexation, with possible biomedical applications, when exploited in the preparation of magnetic resonance imaging contrast agents and radioisotope labeling for *α*‐ and *β*‐therapy. Its structural and electronic characteristics make picolinic acid versatile and able to form stable complexes with various metal ions. The present study reports the synthesis of four picolinic acid derivatives useful for both coordination and bioconjugation, featuring a benzyl‐protected hydroxy group removable via palladium‐catalyzed hydrogenolysis. Chromatography and magnetic resonance assessed the purity, identity, and structural features of the synthesized compounds, ensuring their suitability for the said applications. Single‐crystal X‐ray diffraction was exploited for structure solution of three of them, revealing that the introduction of specific substituents induces changes in both the molecular structure and the crystal packing, driven by a balance between *π*‐stacking and weak nonbonding contacts. Small changes in the pyridine substituents can induce conformational changes in the opposite benzyl ring orientation, spanning from a planar to a perpendicular orientation. The structural analysis, including Hirshfeld surfaces and energetic frameworks calculations, clarified intermolecular interactions contributing to a better understanding of the solid state behavior of the title compounds.

## Graphical abstract

Subtle changes transform crystal structures: synthesis, interactions, and flexibility of novel picolinic acid derivatives with promising biomedical applications, detailed through X‐ray diffraction and conformational modeling.

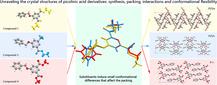

## 1 Introduction

Picolinic acid and its derivatives have increasingly been used as pendant arms in linear or macrocyclic chelators for metal ions complexation with possible application in the biomedical field.^[^ ^1^, ^2^, ^3^, ^4^, ^5^ ^]^ An example could be represented by Gd(III) complexation for application as contrast agents in magnetic resonance imaging.^[^ ^6^ ^]^ Picolinic acid's application has also been extended to polydentate ligands for radioisotopes labeling, as in the case of *α*‐therapy with 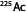 or *β*‐therapy with 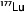. Its peculiar structure combines both carboxylate and pyridine nitrogen coordination in a geometrical configuration that allow the two moieties to form stable 5‐term chelate rings suitable for a stable complexation of larger metals.^[^ ^7^ ^]^ Another important advantage is their versatility: the presence in the same arm of a carboxylate oxygen and a pyridine nitrogen donors gives the picolinic residue the ability to complex soft metals like 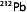 or harder ones as 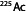,^[^ ^8^ ^]^ according with HSAB theory.^[^ ^9^, ^10^ ^]^ In recent years, these probes have been investigated also for molecular imaging and targeting purposes. By equipping them with an additional functional group, it is possible to perform a bioconjugation with biological vectors like peptides, proteins, or antibodies.^[^ ^11^, ^12^, ^13^ ^]^ These bifunctional probes allow the specific targeting and possible cellular absorption of the drug, reducing the radioactivity dose, and limiting the side effects related to radiation exposure. There are several possible approaches for bioconjugation, evaluating the type of target and the biological vector, such as azido‐alkyne Click‐chemistry bioconjugation,^[^ ^14^ ^]^ NHS acid activation,^[^ ^15^ ^]^ or isothiocyanate coupling to amines.^[^ ^16^ ^]^ Research is always directed toward designing new strategies to obtain pendant arms that are able to adapt to different metal ion complexation and bioconjugation with various biological targets. For this reason, some of the authors developed a picolinic acid derivative with a hydroxy reactive site at the fourth position that acts as a platform ready to be transformed in a reactive moiety for bioconjugation. Specifically, a picolinic derivative with the hydroxy group protected with a benzyl group was synthesized. After the functionalization of the ligand, this benzyl group could be easily removed after a palladium‐catalyzed hydrogenation, leaving the hydroxyl group free to functionalization. In a context of a future approval for the clinical use of a new complex, an industrial scale‐up must be made and so it is important to create a robust, fast, and cheap characterization protocol to monitor each synthetic step. Such monitoring is commonly performed in pharmaceutical companies using X‐ray powder diffraction (XRPD),^[^ ^17^, ^18^, ^19^ ^]^ which outputs patterns that serve as unique fingerprints for each compound. While it is not strictly necessary to know the crystalline structure of a material to identify it in a polycristalline mixture,^[^ ^20^ ^]^ having information about the unit cell, symmetry, and atomic positions undoubtedly simplifies the process and places the user in the best position to carry out further analyses, such as structural fitting of the diffraction pattern through Rietveld refinement. Having access to structural knowledge not only facilitates qualitative analysis but also enhances textural and quantitative analysis,^[^ ^19^, ^21^ ^]^ which can be used to assess process yield. In this work, the synthesis and characterization via HPLC‐UV‐MS, 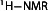, 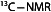, and SC‐XRD and first‐principle modeling of picolinic acid derivatives useful for the synthesis of bifunctional ligands are discussed. Of the four synthesized compounds, three formed crystals suitable for structural resolution by SC‐XRPD data. The crystal packing and the main features of the structures are discussed. Hirshfeld surfaces, the relative 2D fingerprint plots, and the energetic framework calculation allowed a clear visualization and quantitative assessment of intermolecular interactions, highlighting close contacts within the crystal structure. This approach helps in understanding the molecular packing and interactions, which influence the stability and properties of the crystals. Moreover, such conformational flexibility are valuable information to understand what could be the behavior of the molecules in solution. All the reported information will allow for easy and rapid monitoring and verification of the product, being able to enhance the production and, therefore, to open new possibilities in cancer treatment worldwide.

## 2 Experimental Section

**Scheme 1:**
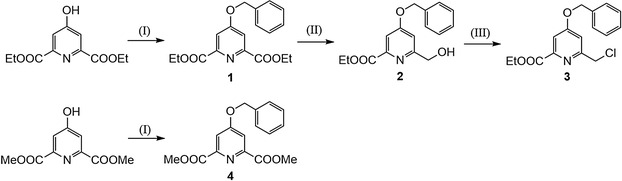
Organic synthesis of the compounds 1 to 4 discussed. (I) = $K_{2}{\text{CO}}_{3}$, benzyl bromide, ACN, reflux 4 h. (II) = EtOH, ${\text{NaBH}}_{4}$, 1 h reflux. (III) = ${\text{SOCl}}_{2}$, DCM, rt, 3 h.

Reagents and solvents were purchased from Sigma‐Aldrich‐Merck and used without further purification. For thin‐layer chromatography and chromatography columns, Merck Silicagel 60 F254 silica (0.063–0.200 mm; 70–230 mesh ASTM) was used. UV light (254 nm), alkaline potassium permanganate was used to visualize the stain in TLC. For HPLC analysis and mass spectra, a coupled HPLC‐MS system equipped with a Waters 1525 binary HPLC pump system using XTerra 3.5 μm (4.6×150 mm) analytical columns was used. A Waters 3100 Mass Detector with ESI+ source and quadrupole was used as a mass analyzer. The 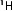 and 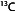 NMR spectra were recorded using a Bruker Avance III 500 MHz spectrometer equipped with a 5 mm PABBO probe and a BVT‐3000 temperature control unit, using tetramethylsilane as reference for chemical changes (*δ* [ppm]). Multiplicities were reported as singlet (*s*), doublet (*d*), triplet (*t*), quartet (*q*), or multiplet (*m*). The samples were prepared in 5 mm NMR tubes by dissolving the compounds in appropriate deuterated solvents. All NMR spectra were reported as Figure S1–S8, Supporting Information. The reaction sequence is reported in **Scheme** **1**.

### 2.1 Synthesis of Diethyl 4‐(Benzyloxy)Pyridine‐2,6‐Dicarboxylate (Compound 1)

Diethyl 4‐(hydroxy)pyridine‐2,6‐dicarboxylate (500 mg, 2.08 mmol) and potassium carbonate (1.72 g, 12.50 mmol) were dissolved in ACN (10 mL). Benzyl bromide was added dropwise, and the reaction was stirred under a nitrogen atmosphere, refluxing for 4 h. The residual carbonate was filtered out, and the solvent was removed under reduced pressure. Crystals suitable for SC‐X‐ray diffraction determination (588 mg) were obtained by dissolving the crude product in hexane and leaving at 4° overnight. Yield: 86%, mp: 40 °C–42 °C.

HPLC‐MS (Water XTerra, 4.6 × 100 mm ((A): $H_{2}O$ + 0.1% TFA, (B): ACN, flow = 1$\text{mL}\;{\text{min}}^{-1}$, from 0 to 12 min: from 10% B to 100%, from 12 min to 15 min 100% B). Rt: 9.63 min.

ESI‐MS (m/z): found 330.4 (M + H+) (calc. for $C_{18}H_{19}{\text{NO}}_{5}\text{:}\;329.13$).


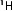
 NMR (500 MHz, ${\text{CDCl}}_{3}$): *δ* = 1.53 (*t*, 4 J = 2.6 Hz, 6H, $[chemistry single bond solid line]{\text{CH}}_{3}$), 4.56 (*q*, 4 J = 2.6 Hz, 4 H, $[chemistry single bond solid line]{\text{CH}}_{2}$), 5.30 (*s*, 2 H, $O[chemistry single bond solid line]{\text{CH}}_{2}[chemistry single bond solid line]\text{Bz}$), 7.45–7.53 (*m*, 5 H, H‐Bz), 7.94 (*s*, 2 H, H‐Py) ppm.


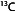
 NMR (125 MHz, ${\text{CDCl}}_{3}$): *δ* = 14.2 (${\text{CH}}_{3}$), 62.4 (${\text{OCH}}_{2}$), 70.7 (Bz—O—CH_2_), 114.6 (C‐Bz), 127.7 (C‐Bz), 128.7 (C‐Bz), 128.8 (C‐Py), 134.7 (C‐Bz), 150.2 (C‐Py), 164.6 (C—O—CH_2_—Bz), 166.6 (C=O) ppm.

### 2.2 Synthesis of Ethyl 4‐(Benzyloxy)‐6‐(Hydroxymethyl)Pyridine‐2‐Carboxylate (Compound 2)

Diethyl 4‐(benzyloxy)pyridine‐2,6‐dicarboxylate (200 mg, 0.608 mmol) was dissolved in EtOH (6 mL), and ${\text{NaBH}}_{4}$ (20 mg, 0.486 mmol) was slowly added to the solution. The reaction was stirred at reflux for 1 h. The crude product was extracted in DCM/$H_{2}O$ and checked by thin‐layer chromatography (5:5 hexane/ethyl acetate, Rf = 0.18). A white powder was obtained (118 mg) and used without further purification. Yield: 67%, mp: 121 °C–123 °C.

HPLC‐MS (Water XTerra, 4.6 × 100 mmol ((A): $H_{2}O$ + 0.1% TFA, (B): ACN, flow = 1$\text{mL}\;{\text{min}}^{-1}$, from 0 to 12 min: from 10% B to 100%, from 12 to 15 min 100% B). Rt: 6.52 min.

ESI‐MS (m/z): found 288.9 (M + H+) (calc. for $C_{16}H_{17}{\text{NO}}_{4}\text{:287}\text{.12}$).


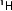
 NMR (500 MHz, ${\text{CDCl}}_{3}$): *δ* = 1.43 (*t*, 4 J = 2.4 Hz, 3 H, $[chemistry single bond solid line]{\text{CH}}_{3}$), 4.32 (*q*, 4 J = 2.4 Hz, 2 H, $-{\text{CH}}_{2}$), 5.15 (*s*, 2 H, $[chemistry single bond solid line]{\text{CH}}_{2}[chemistry single bond solid line]\text{OH}$), 5.25 (*s*, 2 H, $O[chemistry single bond solid line]{\text{CH}}_{2}[chemistry single bond solid line]\text{Bz}$), 7.32‐7.48 (*m*, 5 H, H‐Bz), 7.94 (*s*, 2 H, H‐Py) ppm.


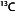
 NMR (125 MHz, ${\text{CDCl}}_{3}$): *δ* = 14.2 (${\text{CH}}_{3}$), 60.4 (${\text{OCH}}_{2}$), 64.5 ($[chemistry single bond solid line]{\text{CH}}_{2}[chemistry single bond solid line]\text{OH}$), 70.9 ($\text{Bz}[chemistry single bond solid line]O[chemistry single bond solid line]{\text{CH}}_{2}$), 114.6 (C‐Bz), 127.0 (C‐Bz), 127.7 (C‐Bz), 128.2 (C‐Py), 136.1 (C‐Bz), 156.4 (OH‐C‐Py), 160.6 ($C[chemistry single bond solid line]O[chemistry single bond solid line]{\text{CH}}_{2}[chemistry single bond solid line]\text{Bz}$), 166.6 (C=O) ppm.

### 2.3 Synthesis of Ethyl 4‐(Benzyloxy)‐6‐(Chloromethyl)Pyridine‐2‐Carboxylate (Compound 3)

Ethyl 4‐(benzyloxy)‐6‐(hydroxymethyl)pyridine‐2‐carboxylate (118 mg, 0.41 mmol) was placed in an ice bath, and 1 mL of ${\text{SOCl}}_{2}$ was added. The reaction was left at room temperature for 3 h. The crude product was neutralized with saturated ${\text{NaHCO}}_{3}$ solution; the solvent was removed under reduced pressure and redissolved in DCM, washing it with 4 × 10 mL $H_{2}O$. The resulting solution was characterized by thin‐layer chromatography (5:5 hexane/ethyl acetate, Rf = 0.71). A yellowish oil containing small crystals was obtained (125 mg). The slow evaporation of this concentrated solution resulted in the formation of larger crystals suitable for SC‐XRD analysis. Yield: 100%, mp: 71 °C–73 °C.

HPLC‐MS (Water XTerra, 4.6 × 100 mm ((A): $H_{2}O$ + 0.1% TFA, (B): ACN, flow = 1$\text{mL}\;{\text{min}}^{-1}$, from 0 to 12 min: from 10% B to 100%, from 12 to 15 min 100% B). Rt: 9.65 min.

ESI‐MS (m/z): found 306.3 (M + H+) (calc for $C_{16}H_{16}{\text{ClNO}}_{3}\text{:305}\text{.08}$).


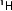
 NMR (500 MHz, ${\text{CDCl}}_{3}$): *δ* = 1.53 (*t*, 4 J = 2.8 Hz, 3 H, $[chemistry single bond solid line]{\text{CH}}_{3}$), 4.56 (*q*, 4 J = 2.8 Hz, 2 H, $[chemistry single bond solid line]{\text{CH}}_{2}$), 4.96 (*s*, 2 H, $[chemistry single bond solid line]{\text{CH}}_{2}[chemistry single bond solid line]\text{Cl}$), 5.30 (*s*, 2 H, $O[chemistry single bond solid line]{\text{CH}}_{2}[chemistry single bond solid line]\text{Bz}$), 7.45‐7.53 (*m*, 5 H, H‐Bz), 7.94 (*s*, 2 H, H‐Py) ppm.


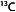
 NMR (125 MHz, ${\text{CDCl}}_{3}$): *δ* = 14.2 (${\text{CH}}_{3}$), 40.9 ($[chemistry single bond solid line]{\text{CH}}_{2}[chemistry single bond solid line]\text{Cl}$), 62.4 (${\text{OCH}}_{2}$), 70.7 ($\text{Bz}[chemistry single bond solid line]O[chemistry single bond solid line]{\text{CH}}_{2}$), 114.6 (C‐Bz), 127.7 (C‐Bz), 128.7 (C‐Bz), 128.8 (C‐Py), 134.7 (C‐Bz), 150.2 ($\text{Cl}[chemistry single bond solid line]{\text{CH}}_{2}[chemistry single bond solid line]\text{Py}$), 164.6 ($C[chemistry single bond solid line]O[chemistry single bond solid line]CH_{2}[chemistry single bond solid line]Bz$), 166.6 (C=O) ppm.

### 2.4 Synthesis of Dimethyl 4‐(Benzyloxy)Pyridine‐2,6‐Dicarboxylate (Compound 4)

Dimethyl 4‐(hydroxy)pyridine‐2,6‐dicarboxylate (500 mg, 2.36 mmol) and potassium carbonate (1.96 g, 14.21 mmol) were dissolved in ACN (10 mL). Benzyl bromide (697 μL, 2.83 mmol was added dropwise, and the reaction was stirred under nitrogen atmosphere, refluxing for 4 h. The residual carbonate is filtered out, and the solvent was removed under reduced pressure. The crude product was characterized by thin‐layer chromatography (5:5 hexane/ethyl acetate, Rf = 0.44) and purified by silica chromatography (7:3 hexane/ethyl acetate). A colorless crystal product suitable for SC‐XRD was obtained by slow evaporation of a concentrated solution of the product in ethyl acetate (547 mg). Yield: 77%, mp: 112 °C–114 °C.

The product was characterized by HPLC‐MS (Water XTerra, 4.6 × 100 mm ((A): $H_{2}O$ + 0.1% TFA, (B): ACN, flow = 1$\text{mL}\;{\text{min}}^{-1}$, from 0 to 12 min: from 10% B to 100%, from 12 min to 15 min 100% B). Rt: 8.44 min.

ESI‐MS (m/z): found 302.6 (M + H+) (calc for $C_{16}H_{15}{\text{NO}}_{5}\text{:301}\text{.10}$).


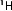
 NMR (500 MHz, ${\text{CDCl}}_{3}$): *δ* = 4.56 (*s*, 6 H, $[chemistry single bond solid line]{\text{CH}}_{3}$), 5.16 (*s*, 2 H, $O[chemistry single bond solid line]{\text{CH}}_{2}[chemistry single bond solid line]\text{Bz}$), 7.45‐7.53 (*m*, 7 H, H‐Bz), 8.55 (*s*, 2 H, H‐Py) ppm.


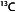
 NMR (125 MHz, ${\text{CDCl}}_{3}$): *δ* = 51.5 (${\text{CH}}_{3}$), 70.9 ($\text{Bz}[chemistry single bond solid line]O[chemistry single bond solid line]{\text{CH}}_{2}$), 112.3 (C‐Bz), 127.7 (C‐Bz), 128.6 (C‐Bz), 128.8 (C‐Py), 136.7 (C‐Bz), 148.2 (C‐Py), 161.8 ($C[chemistry single bond solid line]O[chemistry single bond solid line]{\text{CH}}_{2}[chemistry single bond solid line]\text{Bz}$), 165.9 (C=O) ppm.

### 2.5 SC‐XRD Characterization and Refinement

Single‐crystal X‐ray diffraction (SC‐XRD) data were collected using an Oxford Diffraction (Rigaku) Xcalibur diffractometer with graphite monochromated $\text{Mo}K_{\alpha }$ (*λ* = 0.71073 Å) radiation. Data reduction and absorption corrections were performed exploiting CrysAlisPro 42.49.^[^ ^22^ ^]^ Crystal structure solution was performed by direct methods using ShelXT^[^ ^23^ ^]^ within the Olex2^[^ ^24^ ^]^ GUI. The structural refinement was carried out against $F^{2}$ for all data using the same softwares. All hydrogen atoms on aromatic C—H groups were positioned based on calculated geometric placements and refined as riding atoms on their parent carbon atoms. The isotropic displacement parameter for these hydrogen atoms, $U_{\text{iso}}(H)$, was set to 1.2 times the equivalent isotropic displacement parameter of the corresponding carbon atoms, $U_{\text{eq}}(C)$. Structural visualization and void analysis was carried out with CCDC Mercury 4.0.^[^ ^25^ ^]^ Bond distances and angles tables are reported as Supporting Information (Table S1–S9). Crystallographic information files of the solved crystal structures were deposited to the CCDC database with the numbers: compound 1 CCDC number 2403841, compound 3 CCDC number 2403857, amd compound 4 CCDC number 2403868.

### 2.6 In silico Analyses

Hirshfeld surfaces, fingerprint plots, and energy frameworks were computed through first principle calculations exploiting CrystalExplorer 21.5^[^ ^26^ ^]^ coupled with the Gaussian 16 software^[^ ^27^ ^]^ for the wavefunctions computation. All the analyses were performed with high‐resolution settings and exploited wavefunctions at the DFT CE‐B3LYP level of theory^[^ ^28^ ^]^ with 6‐31G(d,p) basis set. Energy frameworks were represented by the means of cylinders, which size scale was set to 80, with an energy cutoff value of $0\;\text{kJ}\;{\text{mol}}^{-1}$. Interaction energies between each molecule and its surrounding chemical environment were calculated. The lattice energy for each individual molecule was then determined as half the product of the number of symmetry‐equivalent molecules in the cluster and the total interaction energy, according to the method described by Thomas et al.^[^ ^29^ ^]^ Finally, molecular geometric optimization calculations were performed using the B3LYP/6‐31G(d,p) level of theory to compare the conformations of molecules optimized in a vacuum with the asymmetric units of the crystalline structures. Potential energy scans were performed using the Moldraw software^[^ ^30^ ^]^ to investigate the rotational freedom of the benzyl group in the three compounds.

## 3 Results and Discussion

Three out of four substituted 4‐benzyloxypyridines were successfully synthesized and crystallized starting from diethyl (or dimethyl) 4‐hydroxypyridine‐2,6‐dicarboxylate. The synthetic procedures, described in the experimental, allowed to obtain benzyl ether formation, monoreduction of the esters to alcohol which then was reacted with ${\text{SOCl}}_{2}$ to obtain the chloromethyl derivative ready to be used for amine alkylation. Despite the very similar core structure, the three compounds exhibit distinct crystal habits and packing arrangements. The substituents, in position 2 of the pyridine ring for compounds 1–3 and in both positions 2 and 6 of the same ring for compound 4, resulted able to induce different molecular conformations. To highlight these peculiar behaviors, in Section 3.1, the crystal structures are briefly described, followed by an analysis of intermolecular interactions using Hirshfeld surface analysis in Section 3.2 and, finally, in Section 3.3, the differences at both the molecular and crystal packing levels is elucidated, also with the aid of energy framework calculations.

### 3.1 Description of the Crystal Packing

**Figure 1:**
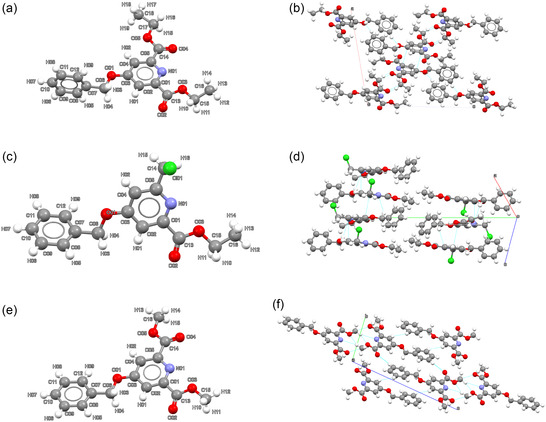
Asymmetric units (a,c,e) and packings (b,d,f) of the three title compounds. From top to bottom: compound 1 (a,b), compound 3 (c,d), and compound 4 (e,f).

The SC‐XRD characterization of compound 1 shows that the compound crystallizes in the monoclinic crystal system within the $P2_{1}/n$ space group. Its cell parameters, reported along with other relevant crystallographic parameters in **Table** **1**, are a=14.992(1) Å, b=7.6172(7) Å, c=16.237(1) Å, and β =100.99(1)°. The structure has one molecule of compound 1 in the asymmetric unit (**Figure** **1**a). The compound presents a 85.45(1)° angle between the pyridine and phenyl ring planes, making the rings nearly orthogonal. *π*‐stacking occurs between symmetry‐related pyridine rings (Figure 1b), with a centroid‐centroid distance of 3.616(5) Å and a N01‐centroid…centroid angle of 104.07(4)°.

**Table 1:** Crystal and structure refinement data for the three title compounds successfully crystallized.

Name	Compound 1	Compound 3	Compound 4
Empirical formula	$C_{18}H_{19}{\text{NO}}_{5}$	$C_{16}H_{16}{\text{ClNO}}_{3}$	$C_{16}H_{15}{\text{NO}}_{5}$
Formula weight [g mol^−1^]	329.34	305.75	301.29
Temperature [K]	293(2)	293(2)	293(2)
Crystal system	Monoclinic	Monoclinic	Triclinic
Space group	$P2_{1}/n$	$P2_{1}/c$	P−1
a [Å]	14.992(1)	8.0062(1)	5.6869(8)
b [Å]	7.6172(7)	29.304(5)	7.3896(1)
c [Å]	16.237(1)	7.3393(1)	17.9905(1)
*α* [°]	90	90	97.191(1)
*β* [°]	100.99(1)	109.72(2)	91.563(9)
*γ* [°]	90	90	106.046(1)
Volume [Å^3^]	1820.3(4)	1620.9(5)	719.34(1)
Average atomic volume [Å^3^]	18.96	19.30	16.35
Z	4	4	2
$\rho_{\text{calc}}$ [g cm^−3^]	1.202	1.253	1.391
μ mm^−1^	0.088	0.244	0.104
F(000)	696.0	640.0	316.0
Radiation [Å]	$\text{Mo}K_{\alpha }$ (*λ* = 0.71073)	$\text{Mo}K_{\alpha }$ (*λ* = 0.71073)	$\text{Mo}K_{\alpha }$ (*λ* = 0.71073)
2*θ* range for data collection [°]	6.248 to 56.494	6.058 to 56.990	7.996 to 57.058
Goodness‐of‐fit on F2	0.988	1.017	1.164
Final R indexes	R1 = 0.0763, wR2 = 0.2022	R1 = 0.0925, wR2 = 0.3294	R1 = 0.0711, wR2 = 0.1753

Steric constraints with symmetry‐equivalent counterparts prevent the benzene ring in compound 1 from forming optimal *π*‐stacking interactions. Instead, structural consolidation is achieved through many weak interactions, including H08… O01 (*d* = 2.592(1) Å) and a *π*‐H interaction with H09 (*d* =3.153(4) Å). Due to such intermolecular contacts, the angle between the planes of two facing benzene rings is equal to 62.77(4)°. N01 interacts with H16 (*d* = 2.611(4) Å), while O04 forms contacts with H15, H16, and H17 (*d* = 2.793(1) Å, *d* = 2.893(1) Å, and *d* = 2.910(1) Å, respectively). Noteworthy, due to the inefficiency of benzene rings in facilitating *π*‐stacking interactions, voids of ≈$117.8{Å}^{3}$ form around the phenyl ring, accounting for 6.5% of the total unit cell volume.

Compound 3 crystallizes in the monoclinic system within the $P2_{1}/c$ space group (Figure 1c,d). Cell parameters, reported in Table 1, are a=8.0062(1) Å, b=29.304(5) Å, c=7.3393(1) Å, and 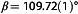. The asymmetric unit contains a single molecule of compound 3 (Figure 1c), adopting a configuration with a 62.59(1)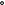 angle between the pyridine and benzyl ring planes. The C8–Cl1 bond is oriented almost perpendicular to the pyridine ring plane [*τ*(N01–C05–C14–Cl01) = −86.6(5)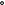], resulting in electrostatic interactions with the partially positively charged hydrogen H11 (a T‐like interaction). Additionally, nitrogen N01 interacts electrostatically with H03, while the carbonyl oxygen forms contacts with H02 and H15 from a symmetry‐related neighboring molecule, facilitating close positioning of the pyridine rings. These rings show *π*‐stacking with a centroid‐to‐centroid distance of 3.817(1) Å and an interplanar angle of 108.06(1)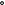, consistent with efficient *π*‐*π* stacking. However, a less compact packing is observed with columns of molecules separated by less dense zones (Figure 1d), as also confirmed by the large volume/atom value (Table 1). Such less dense packing in the intercolumn area is caused by the benzyl groups, which cannot fully exploit *π*‐stacking due to packing constraints, resulting in a centroid‐centroid distance of 4.874(3) Å and an inter‐centroid angle of 111.73(1)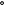.

Compound 4 crystallizes in the triclinic crystal system, within the $P\bar{1}$ space group. Its cell parameters are a=5.6869(8) Å, b=7.3896(1) Å, c=17.9905(1) Å, 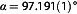, 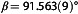, and 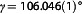. The asymmetric unit contains a single molecule in a nearly planar conformation, with an inter‐ring plane angle of 9.56(1)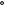. Thermal ellipsoid analysis confirms that this angle is within the range of typical thermal motion, and thus the moiety can be considered definitely planar. The molecules arrange in parallel layers, separated by 3.391(4) Å. However, *π*‐stacking is inefficient for both aromatic rings due to the inversion center, resulting in unfavorable ring facing.

The structure is consolidated by extensive weak nonbonding interactions between adjacent layers, similarly to compound 1. For example, H04 interacts with the benzene ring of an adjacent layer at a short H04…centroid distance of 2.568(4) Å and an angle ∠(C07‐centroid…H04) of 92.61(1)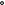.

Within each layer, weak interactions form an electrostatic network between in‐plane symmetry‐related molecules (Figure 1e,f). Notable contacts include N01…H12 (*d* = 2.635(2) Å) and interactions of H12 with O03 and O04 atoms (*d* = 2.638(1) Å and *d* = 2.729(1) Å, respectively). The benzene positioning ring is further consolidated by a H06…O02 interaction (*d* = 2.487(5) Å), contributing to its coplanarity within the molecular structure.

All the described structures exhibit less efficient packing compared to those reported in the scientific literature for analogous compounds.^[^ ^31^, ^32^ ^]^ The main contributing factor, particularly when comparing the structures presented in this work with those reported by Rabe and coworkers,^[^ ^31^ ^]^ appears to be the presence of the benzyloxy substituent at the 4‐position of the pyridine ring. Structures such as CCDC 818684 also display disorder located on the same ring. A second reason lies in the impossibility, observed in the two nonplanar structures (compound 1 and compound 3), to establish synthons or weaker in‐plane interactions, a factor that is typically crucial for achieving compact and efficient packing, as seen in the case of dimethyl pyridine‐2,6‐dicarboxylate^[^ ^33^, ^34^ ^]^ or in the case of pyridine‐2,6‐dicarboxylic acid.^[^ ^35^, ^36^ ^]^

### 3.2 Hirshfeld Surface Analysis

The analysis of Hirshfeld surfaces and their corresponding 2D fingerprint plots offers valuable insights into the nature and distribution of intermolecular interactions within the crystalline lattice, providing a comparative framework to understand the structural characteristics and interaction patterns of compounds 1, 3, and 4. Compound 1 exhibits a nonuniform color tone on the Hirshfeld surface. This is mainly due to two factors: the first is the presence of close contacts, as described in the previous section, which connect the molecules with weak forces; the second factor is the formation of voids within the structure. These opposite behaviors are confirmed by the intermediate volume/atom of compound 3 (Table 1) and can be recognized on the Hirshfeld surface by the bright blue regions and are located in the volume portion where two symmetry‐related phenyl rings and two symmetry‐related ethyl groups (C10–C16) face each other. The volume defined by these four terminal groups corresponds to ≈6.5% of the total unit cell volume. Such void region contributes to the elongation along the bisector direction of the 2D fingerprint plot of the structure: H…H and C…H interactions extend up to distances of $d_{i}=3.0,d_{e}=2.8$. The short H…H contacts reach $d_{i}=1.2,d_{e}=1.2$, followed by O…H contacts ($d_{i}=1.4,d_{e}=1.1$) and N…H contacts ($d_{i}=1.5,d_{e}=1.1$). The H…H interactions represent the highest percentage among the three structures, at 47% of the total, while the C…C interactions, attributed to *π* stacking, makes up only 1.8% of the total, the lowest among the three structures, despite a minimum distance of $d_{i}=1.7,d_{e}=1.7$.

**Figure 2:**
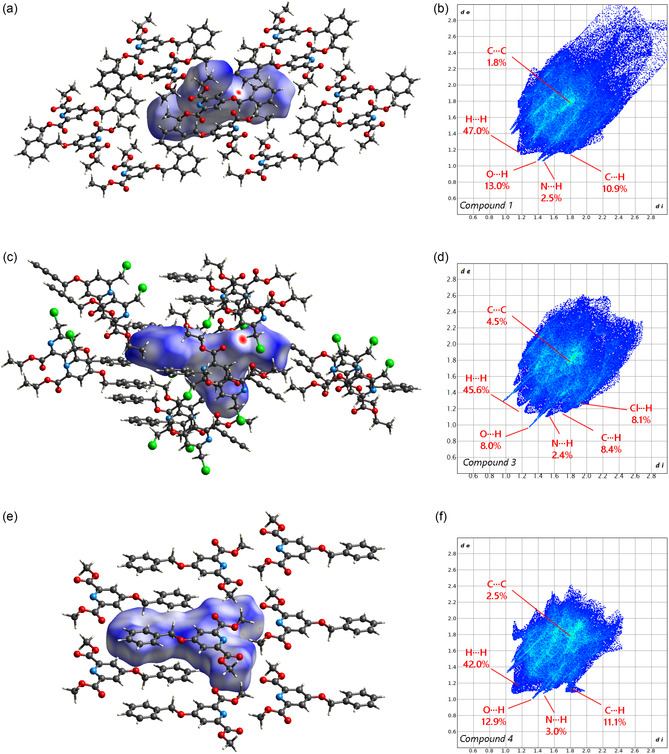
Calculated Hirshfeld surfaces (a,c,e) and 2D fingerprint plots (b,d,f) of the three title compounds. From top to bottom: compound 1 (a,b),compound 3 (c,d), and compound 4 (e,f).

The Hirshfeld surface of compound 3, highlighted for $d_{\text{norm}}$, reveals short contacts within the structure, specifically O02… H16 (*d* = 2.281(1) Å) and, to a lesser extent, O02…H02 (*d* = 2.491(1) Å). The surface appears relatively uniform, except for the central region of the pyridine ring, which displays a lighter tone, an indication of *π*‐stacking, as described in the previous section. The 2D fingerprint plot is symmetrical with respect to the plot's bisector, due to the presence of only one molecule in the asymmetric unit. The plot takes the shape of an ellipsoid, suggesting highly consistent distances and reinforcing the interpretation of the surface. The short contacts are highlighted by spikes at $d_{i}=1.3,d_{e}=0.95$, corresponding to the oxygen‐hydrogen contacts previously described. Notably, 45.6% of the contacts on the Hirshfeld surface occur between hydrogen atoms classified as internal and external, confirming that weak forces dominate the interactions within the structure. The spike at $d_{i}=1.1,d_{e}=1.5$ corresponds to an N…H contact. In the fingerprint plot, the light blue central area of the ellipse (at $d_{i}=1.8,d_{e}=1.8$) corresponds to *π*‐stacking between the two pyridine rings, while the less efficient stacking of phenyl rings appears at $d_{i}=2.2,d_{e}=2.2$ (**Figure** **2**).

Compound 4 displays a relatively homogeneous surface color, with lighter areas corresponding to the benzene ring, where intralayer contacts form between the ring and hydrogen atoms H03 and H04, and to the region around the nitrogen atom of the pyridine ring, where additional intralayer contacts (N01…H10) are established. The portion of the surface perpendicular to the ring planes confirms previous observations, as it shows a region with frequent short contacts, forming the network of interactions that primarily consolidate the molecules within the plane of the aromatic rings. The 2D fingerprint plot shows a less homogeneous contour compared to the well‐defined ellipsoid of compound 3, while it appears more compact. The shortest contact, represented by a large spike at $d_{i}=1.1,d_{e}=1.1$, corresponds to hydrogen‐hydrogen interactions, which globally account for 42% of the interactions. The contact represented by a spike at $d_{i}=1.3,d_{e}=1.0$ is attributed to O…H interactions, which are close to the spike at $d_{i}=1.5,d_{e}=1.1$ corresponding to N…H interactions. The “wing‐shaped” portion of the plot at $d_{i}=1.7,d_{e}=1.1$ is due to C…H interactions present within the structure. As expected, C…C interactions due to *π*‐stacking effects are located around $d_{i}=1.8,d_{e}=1.8$, and these interactions constitute a smaller percentage of the total interactions (${\text{C…C}}_{\text{Compound3}}$ = 4.5%, ${\text{C…C}}_{\text{Compound2}}$ = 2.5%).

**Figure 3:**
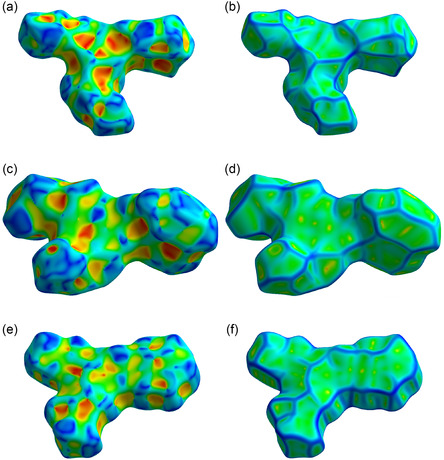
Shape index surfaces (a,c,e) and curvedness surfaces (b,d,f) of the three title compounds. From top to bottom: compound 1 (a,b), compound 3 (c,d), and compound 4 (e,f).

The Hirshfeld surface analysis further corroborates the observed packing differences. As shown in **Figure** **3**, the shape index maps of compounds 1 to 4 do not display the characteristic red and blue triangular patches typically associated with π−π stacking interactions, in contrast to structurally related systems. This absence is consistent with the nonplanarity induced by the benzyloxy substituent at position 4 of the pyridine ring, which prevents efficient face‐to‐face aromatic contacts. In addition, the curvedness maps reveal a lack of extended flat regions, further confirming the geometrical constraints that limit directional interactions within the molecular layers. These surface features align well with the reduced packing efficiency and the increased molecular disorder observed in the crystal structures, in particular in the structures of compounds 1 and 3.

The Hirshfeld surface and fingerprint plot analyses provide a detailed understanding of the intermolecular interactions in the solid state. Specifically, the high percentages of H…H and O…H interactions observed in compounds 1, 3, and 4 suggest a tendency to form weak hydrogen bonds, which which are significant for understanding the molecular packing and stability of these compounds. Similarly, the observed π−π interactions, more or less efficient depending on the sample, are important for the overall molecular arrangement and can influence the structural properties in the solid state.

### 3.3 Energetic Considerations: Conformation, Energy Frameworks and Lattice Energy

**Figure 4:**
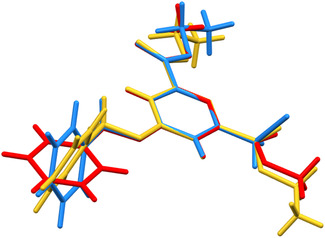
Superposition of the asymmetric units of the title compounds. Yellow: compound 1; blue: compound 3; red: compound 4.

In **Figure** **4**, the asymmetric units of the three title compounds are overlaid, aligned by their pyridine rings. The substituents on the pyridine ring display consistent superimposability with minor deviations attributable to thermal motion, suggesting that the molecule is more stable when the pyridine substituents adopt a near‐coplanar conformation. The most notable variation is observed in the rotation of the benzyl moiety around the methyl–phenyl bond axis. Among the three crystal structures, the benzyl ring adopts varying tilt angles relative to the pyridine ring plane. For compound 4, a nearly planar conformation enables a more compact packing arrangement, as reflected in Table 1 by the average atomic volume ($16.35{Å}^{3}$).

In **Table** **2**, the torsion angles of the benzyl terminal are reported before and after geometry optimization using Gaussian 16. In the second part of the table, the initial model was modified to adopt a fully planar conformation to verify the convergence of the calculations toward the torsional angles identified in the first run. It is evident that in all three molecules, the benzyl groups converge to an angle of ≈90° relative to the plane of the pyridine ring, when an isolated molecule is considered. A potential energy scan on the C08‐C07‐C06‐O01 torsion angle has been performed (Figure S12–S14, Supporting Information), confirming that, despite minor differences in the energy barrier, the most stable conformation for the isolated molecule shows a torsion angle of the benzyl group close to 90° with respect to the pyridine plane, in agreement first principle calculations (Table 2). The energy barriers between the energy minima are indeed small (about less than $20\text{kJ}\;{\text{mol}}^{-1}$), and this explain how the crystal packing (showing much larger lattice energies, see **Table** **3**) is able to select the more convenient torsion angle in each structure (Table 2). In fact, the “perpendicular” configuration is observed in the solid state only for compound 1. For the other two compounds, packing interactions evidently impose the benzyl rotation toward a more planar configuration, highlighting the importance of intermolecular interactions, as described in the previous section.

**Table 2:** Experimental and calculated torsion angles of the benzyl ring (C08‐C07‐C06‐O01). The torsion angle is determined by the sequence of atoms starting from the bridging oxygen, proceeding through the connecting atoms, and ending at the carbon in the 2‐position of the benzyl ring. Two starting models were used: the molecule's configuration within the asymmetric unit and a fully planar starting model.

Name	Starting model	Starting torsion angle [°]	Final torsion angle [°]
Compound 1	Asym. unit	85.3(5)	89.4
Compound 3	Asym. unit	64.8(8)	92.4
Compound 4	Asym. unit	6.2(4)	89.7
Compound 1	Planar	0	89.7
Compound 3	Planar	0	92.5
Compound 4	Planar	0	89.6

**Table 3:** Calculated lattice energies for the title compounds at the DFT CE‐B3LYP/6‐31 G(d,p) level of theory.

Name	Total lattice energy
Compound 1	−158 kJ mol^−1^
Compound 3	−132 kJ mol^−1^
Compound 4	−163 kJ mol^−1^

**Figure 5:**
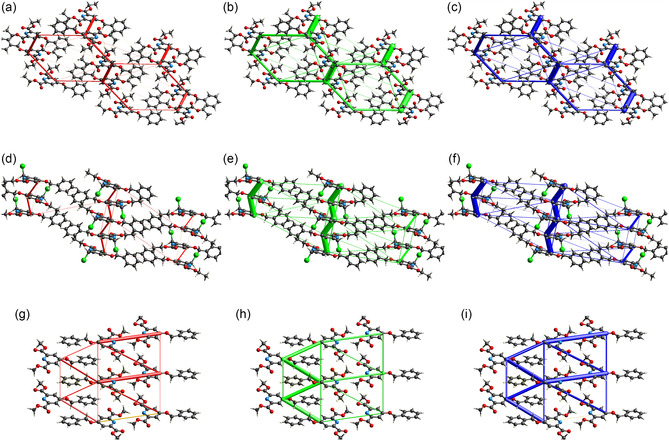
Energy frameworks of the three title compounds: Coulomb energy (a,d,g), dispersion energy (b,e,h), total energy (c,f,i). From top to bottom: compound 1 (a,b,c), compound 3 (d,e,f), and compound 4 (g,h,i).

In **Figure** **5**, the energy framework graphs for the three title compounds are presented. Each row of the figure matrix corresponds to a different compound, in the order compound 1, compound 3, and compound 4, while each column represents a different type of interaction: column 1 (red cylinders) for Coulomb energy, column 2 (green cylinders) for dispersion energy, and column 3 (blue cylinders) for total energy.

In Figure 5a–c, compound 1 shows energy directions predominantly connecting the pyridine rings. The Coulomb energy cylinders are concentrated in the region of the unit cell with a higher number of heteroatoms, as expected. Dispersion energy is more prominently represented along the direction of pyridine *π*‐stacking, and, to a lesser extent, along the direction connecting two symmetrically equivalent pyridines within the same plane. The total energy framework reveals preferential energy directions (larger cylinders), with less favored regions corresponding to areas dominated by hydrocarbons that lack heteroatoms.

Compound 3 exhibits interactions that develop along a plane parallel to the cell edges *a* and *c*. In particular, the strongest Coulomb and dispersion energies connect the pyridine rings of different molecular layers. From the views shown in Figure 5d–f, it is evident that the cylinders representing each energy type are located near the heteroatoms, rather than in the purely hydrocarbon portion of the unit cell.

Compound 4 (Figure 5g–i), in accordance with the observations made in previous sections, exhibits Coulomb energy cylinders along the molecular layers, particularly along the direction connecting two pyridine rings that are equivalent by symmetry and linked by weak interactions. This confirms that H12 is responsible for a kind of face‐to‐face in‐plane interaction between two molecules facing each other due to its interactions with O03, O04, and N01. On the other hand, the dispersion energies connect the molecules more strongly along intralayer directions rather than in‐plane, indicating that they are primarily associated with interactions involving extraplanar hydrogens and the small portion of *π* interaction that can be established. The total energy indicates that the crystal is essentially isotropic in the distribution of the energy framework (Figure 5i).

The lattice energy calculation was performed as outlined in the work by ref. [29] and in previous studies by some of the authors of this paper.^[^ ^37^, ^38^, ^39^ ^]^ The results, shown in Table 3 and obtained with a DFT CE‐B3LYP/6‐31G(d,p) level of theory, indicate compound 4 as the most energetically stable, with a lattice energy value of $-163\;\text{kJ}\;{\text{mol}}^{-1}$, confirmed by the smaller volume/atom (Table 1), the absence of local voids, and an optimal packing of benzyl moieties. Noteworthy, it shows the lowest symmetry ($P\bar{1}$ space group) and a framework enriched with both coplanar intermolecular interactions and interactions spanning multiple molecular layers. The compound 1 and compound 3 structures follow, with lattice energy values of −158 and −132 $\text{kJ}\;{\text{mol}}^{-1}$, respectively. The wave‐like packing of compound 3 (Figure 5d–f) is the less efficient, because of the larger distance between columns of molecules (Figure 1d). Compound 1 has intermediate stability, because of the concurrence of voids and good van der Walls interaction.

The correlation between the calculated lattice energies and the intermolecular interactions within the crystals suggests that the more compact compounds, enriched with electrostatic and *π*‐stacking interactions, may exhibit thermodynamic stability even in solution. This is particularly relevant for the design of ligands intended for biological contexts, where complex stability and predictable interactions with molecular targets are essential. Therefore, the structural and energetic data not only provide insights into the molecular and crystal structures but also help predicting the behaviors of these derivatives in their applications.

## 4 Conclusion

The present study successfully outlined the synthesis, characterization, and structural analysis of four picolinic acid derivatives, with three of them crystallizing into distinct and well‐defined structures. Advanced characterization techniques, including, HPLC‐MS, 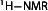, 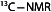, and SC‐XRD, were exploited to confirm the structure of the products and assess reaction yields. The Hirshfeld surface analysis and energetic frameworks provided detailed insights into intermolecular interactions, revealing the role of weak forces, hydrogen bonding, and *π*‐stacking in stabilizing the crystal packing.

Among the compounds studied, compound 4 exhibited the most compact packing and lowest lattice energy, reflecting its superior stability, which is likely to translate into favorable solution properties, also thanks to the flexibility of the benzyl group, able to adapt to the environment, as demonstrated by the different molecular conformations adopted in the three solved crystal structures. These results are pivotal for the future design of bifunctional ligands tailored for bioconjugation and metal complexation, particularly in biomedical fields such as cancer therapy and molecular imaging.

Future work will focus on developing a bifunctional chelate using compound 3 and a molecular imaging probe by conjugation to a specific biological vector. Moreover, the functionalization of these derivatives will allow to explore their potential as versatile ligands for biological applications. Additionally, further studies will aim to correlate the solid‐state properties of these compounds with their solution behavior and biological activity, ensuring their suitability for clinical applications.

**Scheme 2:**
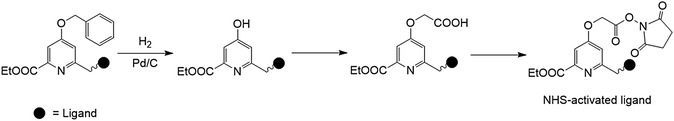
Hypothetical synthesis scheme for the bioconjugation of the functionalized picolinic acid derivative.

To reach this goal, a possible approach is reported in **Scheme** **2** where the benzyl protection is removed with a Pd/C hydrogenation, and the hydroxy functionalization converted for example into an oxyacetic acid group and so into an N‐hydroxysuccinimide activated acid.

## Conflict of Interest

The authors declare no conflict of interest.

## Supporting information

Supplementary Material

## Data Availability

The data that support the findings of this study are available from the corresponding author upon reasonable request.
