# Supplementary material for: Unraveling the Crystal Structures of Picolinic Acid Derivatives: Synthesis, Packing, Interactions, and Conformational Flexibility
Source: ChemistryOpen. 2025 May 2;14(10):e202500197. doi: 10.1002/open.202500197 (PMC12518053; doi:10.1002/open.202500197)
Supplement: Supplementary file 1 — Supplementary Material [file OPEN-14-e202500197-s001.pdf]

Unraveling the Crystal Structures of Picolinic Acid Derivatives: Synthesis, Packing, Interactions and Conformational Flexibility

Sara Camorali[a], Lorenzo Tei[a], Marco Milanesio[a], Mattia Lopresti*[a]

[a]*Dipartimento di Scienze e Innovazione Tecnologica, Universit`a del Piemonte Orientale, Viale Teresa Michel, 22, Alessandria, 15121, Italy*

Table S1: Bond lengths for Compound 1.

Number	Atom1	Atom2	Cyclicity	Length
1	O1	C3	acyclic	1.331(5)
2	O1	C7	acyclic	1.457(4)
3	O2	C4	acyclic	1.350(4)
4	O2	C8	acyclic	1.443(4)
5	O3	C3	acyclic	1.193(6)
6	O4	C6	acyclic	1.320(5)
7	O4	C15	acyclic	1.465(6)
8	O5	C6	acyclic	1.199(5)
9	N2	C1	cyclic	1.337(4)
10	N2	C2	cyclic	1.331(4)
11	C1	C3	acyclic	1.514(5)
12	C1	C10	cyclic	1.377(5)
13	C2	C5	cyclic	1.393(5)
14	C2	C6	acyclic	1.503(6)
15	C4	C5	cyclic	1.388(5)
16	C4	C10	cyclic	1.391(5)
17	C5	H5	acyclic	0.93
18	C7	H7A	acyclic	0.97
19	C7	H7B	acyclic	0.969
20	C7	C11	acyclic	1.501(6)
21	C8	H8A	acyclic	0.97
22	C8	H8B	acyclic	0.969
23	C8	C9	acyclic	1.493(6)
24	C9	C12	cyclic	1.370(7)
25	C9	C14	cyclic	1.349(6)
26	C10	H10	acyclic	0.93
27	C11	H11A	acyclic	0.96

28	C11	H11B	acyclic	0.96
29	C11	H11C	acyclic	0.96
30	C12	H12	acyclic	0.93
31	C12	C18	cyclic	1.380(8)
32	C13	H13	acyclic	0.93
33	C13	C14	cyclic	1.373(7)
34	C13	C17	cyclic	1.359(9)
35	C14	H14	acyclic	0.93
36	C15	H15A	acyclic	0.97
37	C15	H15B	acyclic	0.971
38	C15	C16	acyclic	1.456(7)
39	C16	H16A	acyclic	0.96
40	C16	H16B	acyclic	0.96
41	C16	H16C	acyclic	0.961
42	C17	H17	acyclic	0.93
43	C17	C18	cyclic	1.363(7)
44	C18	H18	acyclic	0.93

Table S2: Angle measurements for Compound 1.

Number	Atom1	Atom2	Atom3	Angle
1	C3	O1	C7	115.6(3)
2	C4	O2	C8	117.9(3)
3	C6	O4	C15	115.9(3)
4	C1	N2	C2	116.4(3)
5	N2	C1	C3	115.8(3)
6	N2	C1	C10	123.7(3)
7	C3	C1	C10	120.5(3)
8	N2	C2	C5	124.6(3)
9	N2	C2	C6	117.9(3)
10	C5	C2	C6	117.5(3)
11	O1	C3	O3	125.1(4)
12	O1	C3	C1	111.3(3)
13	O3	C3	C1	123.6(3)
14	O2	C4	C5	125.7(3)
15	O2	C4	C10	116.4(3)
16	C5	C4	C10	117.9(3)
17	C2	C5	C4	118.0(3)
18	C2	C5	H5	121
19	C4	C5	H5	121.1
20	O4	C6	O5	124.7(4)
21	O4	C6	C2	112.7(3)
22	O5	C6	C2	122.5(4)
23	O1	C7	H7A	110.3
24	O1	C7	H7B	110.4

25	O1	C7	C11	106.8(3)
26	H7A	C7	H7B	108.6
27	H7A	C7	C11	110.3
28	H7B	C7	C11	110.4
29	O2	C8	H8A	110.3
30	O2	C8	H8B	110.3
31	O2	C8	C9	107.1(3)
32	H8A	C8	H8B	108.6
33	H8A	C8	C9	110.2
34	H8B	C8	C9	110.3
35	C8	C9	C12	121.0(4)
36	C8	C9	C14	121.2(4)
37	C12	C9	C14	117.9(4)
38	C1	C10	C4	119.4(3)
39	C1	C10	H10	120.3
40	C4	C10	H10	120.3
41	C7	C11	H11A	109.4
42	C7	C11	H11B	109.4
43	C7	C11	H11C	109.4
44	H11A	C11	H11B	109.5
45	H11A	C11	H11C	109.5
46	H11B	C11	H11C	109.5
47	C9	C12	H12	119.6
48	C9	C12	C18	120.8(4)
49	H12	C12	C18	119.6
50	H13	C13	C14	119.5
51	H13	C13	C17	119.6
52	C14	C13	C17	120.9(5)
53	C9	C14	C13	121.5(4)
54	C9	C14	H14	119.3
55	C13	C14	H14	119.2
56	O4	C15	H15A	109.9
57	O4	C15	H15B	109.8
58	O4	C15	C16	109.1(4)
59	H15A	C15	H15B	108.2
60	H15A	C15	C16	109.9
61	H15B	C15	C16	109.9
62	C15	C16	H16A	109.5
63	C15	C16	H16B	109.5
64	C15	C16	H16C	109.4
65	H16A	C16	H16B	109.5
66	H16A	C16	H16C	109.5
67	H16B	C16	H16C	109.4
68	C13	C17	H17	121
69	C13	C17	C18	118.1(5)
70	H17	C17	C18	120.9
71	C12	C18	C17	120.7(5)

72	C12	C18	H18	119.7
73	C17	C18	H18	119.6

Table S3: Torsion angle measurements for Compound 1.

Number	Atom1	Atom2	Atom3	Atom4	Torsion
1	C7	O1	C3	O3	-1.5(5)
2	C7	O1	C3	C1	177.5(3)
3	C3	O1	C7	H7A	51.9
4	C3	O1	C7	H7B	-68.1
5	C3	O1	C7	C11	171.9(3)
6	C8	O2	C4	C5	0.7(5)
7	C8	O2	C4	C10	-179.9(3)
8	C4	O2	C8	H8A	59.8
9	C4	O2	C8	H8B	-60.1
10	C4	O2	C8	C9	179.8(3)
11	C15	O4	C6	O5	-1.1(6)
12	C15	O4	C6	C2	176.8(3)
13	C6	O4	C15	H15A	-48.2
14	C6	O4	C15	H15B	70.8
15	C6	O4	C15	C16	-168.7(4)
16	C2	N2	C1	C3	-179.0(3)
17	C2	N2	C1	C10	0.2(5)
18	C1	N2	C2	C5	-0.1(5)
19	C1	N2	C2	C6	179.9(3)
20	N2	C1	C3	O1	-168.7(3)
21	N2	C1	C3	O3	10.3(5)
22	C10	C1	C3	O1	12.1(5)
23	C10	C1	C3	O3	-168.9(4)
24	N2	C1	C10	C4	-0.3(5)
25	N2	C1	C10	H10	179.7
26	C3	C1	C10	C4	178.9(3)
27	C3	C1	C10	H10	-1.2
28	N2	C2	C5	C4	0.1(5)
29	N2	C2	C5	H5	179.9
30	C6	C2	C5	C4	-180.0(3)
31	C6	C2	C5	H5	-0.1
32	N2	C2	C6	O4	17.3(5)
33	N2	C2	C6	O5	-164.7(4)
34	C5	C2	C6	O4	-162.7(3)
35	C5	C2	C6	O5	15.3(6)
36	O2	C4	C5	C2	179.3(3)
37	O2	C4	C5	H5	-0.6
38	C10	C4	C5	C2	-0.1(5)
39	C10	C4	C5	H5	-179.9
40	O2	C4	C10	C1	-179.3(3)
41	O2	C4	C10	H10	0.8

42	C5	C4	C10	C1	0.2(5)
43	C5	C4	C10	H10	-179.8
44	O1	C7	C11	H11A	-55.3
45	O1	C7	C11	H11B	-175.3
46	O1	C7	C11	H11C	64.7
47	H7A	C7	C11	H11A	64.6
48	H7A	C7	C11	H11B	-55.4
49	H7A	C7	C11	H11C	-175.4
50	H7B	C7	C11	H11A	-175.3
51	H7B	C7	C11	H11B	64.7
52	H7B	C7	C11	H11C	-55.3
53	O2	C8	C9	C12	-95.1(5)
54	O2	C8	C9	C14	85.3(5)
55	H8A	C8	C9	C12	24.9
56	H8A	C8	C9	C14	-154.6
57	H8B	C8	C9	C12	144.9
58	H8B	C8	C9	C14	-34.7
59	C8	C9	C12	H12	0.3
60	C8	C9	C12	C18	-179.7(4)
61	C14	C9	C12	H12	179.8
62	C14	C9	C12	C18	-0.1(7)
63	C8	C9	C14	C13	179.0(4)
64	C8	C9	C14	H14	-1
65	C12	C9	C14	C13	-0.6(7)
66	C12	C9	C14	H14	179.4
67	C9	C12	C18	C17	0.9(8)
68	C9	C12	C18	H18	-179
69	H12	C12	C18	C17	-179.1
70	H12	C12	C18	H18	1
71	H13	C13	C14	C9	-179.5
72	H13	C13	C14	H14	0.5
73	C17	C13	C14	C9	0.5(8)
74	C17	C13	C14	H14	-179.5
75	H13	C13	C17	H17	0.3
76	H13	C13	C17	C18	-179.7
77	C14	C13	C17	H17	-179.7
78	C14	C13	C17	C18	0.3(8)
79	O4	C15	C16	H16A	58.2
80	O4	C15	C16	H16B	-61.9
81	O4	C15	C16	H16C	178.2
82	H15A	C15	C16	H16A	-62.3
83	H15A	C15	C16	H16B	177.6
84	H15A	C15	C16	H16C	57.6
85	H15B	C15	C16	H16A	178.6
86	H15B	C15	C16	H16B	58.5
87	H15B	C15	C16	H16C	-61.4
88	C13	C17	C18	C12	-1.0(8)

89	C13	C17	C18	H18	179
90	H17	C17	C18	C12	179
91	H17	C17	C18	H18	-1

Table S4: Bond lengths for Compound 3.

Number	Atom1	Atom2	Cyclicity	Length
1	Cl01	C00D	acyclic	1.741(7)
2	O002	C009	acyclic	1.356(7)
3	O002	C00C	acyclic	1.440(6)
4	O003	C008	acyclic	1.333(7)
5	O003	C00F	acyclic	1.458(7)
6	O004	C008	acyclic	1.212(5)
7	N005	C007	cyclic	1.328(6)
8	N005	C00A	cyclic	1.347(6)
9	C006	H006	acyclic	0.93
10	C006	C007	cyclic	1.379(7)
11	C006	C009	cyclic	1.385(6)
12	C007	C008	acyclic	1.493(6)
13	C009	C00B	cyclic	1.376(7)
14	C00A	C00B	cyclic	1.367(8)
15	C00A	C00D	acyclic	1.507(7)
16	C00B	H00B	acyclic	0.93
17	C00C	H00A	acyclic	0.97
18	C00C	H00C	acyclic	0.97
19	C00C	C00E	acyclic	1.481(8)
20	C00D	H00D	acyclic	0.97
21	C00D	H00E	acyclic	0.971
22	C00E	C00G	cyclic	1.39(1)
23	C00E	C00J	cyclic	1.36(1)
24	C00F	H00F	acyclic	0.97
25	C00F	H00G	acyclic	0.97
26	C00F	C00H	acyclic	1.462(9)
27	C00G	H00H	acyclic	0.93
28	C00G	C00K	cyclic	1.37(1)
29	C00H	H00I	acyclic	0.96
30	C00H	H00J	acyclic	0.96
31	C00H	H00K	acyclic	0.96
32	C00I	H00L	acyclic	0.93
33	C00I	C00K	cyclic	1.35(2)
34	C00I	C00L	cyclic	1.38(3)
35	C00J	H00M	acyclic	0.93
36	C00J	C00L	cyclic	1.39(1)
37	C00K	H00N	acyclic	0.93
38	C00L	H00O	acyclic	0.93

Table S5: Angle measurements for Compound 3.

Number	Atom1	Atom2	Atom3	Angle
1	C009	O002	C00C	118.5(4)
2	C008	O003	C00F	116.8(4)
3	C007	N005	C00A	115.2(4)
4	H006	C006	C007	121.5
5	H006	C006	C009	121.4
6	C007	C006	C009	117.1(4)
7	N005	C007	C006	125.8(4)
8	N005	C007	C008	116.8(4)
9	C006	C007	C008	117.4(4)
10	O003	C008	O004	122.4(5)
11	O003	C008	C007	113.9(4)
12	O004	C008	C007	123.6(5)
13	O002	C009	C006	124.9(5)
14	O002	C009	C00B	116.3(5)
15	C006	C009	C00B	118.8(5)
16	N005	C00A	C00B	123.9(5)
17	N005	C00A	C00D	116.2(4)
18	C00B	C00A	C00D	120.0(5)
19	C009	C00B	C00A	119.2(5)
20	C009	C00B	H00B	120.5
21	C00A	C00B	H00B	120.3
22	O002	C00C	H00A	110.1
23	O002	C00C	H00C	110.1
24	O002	C00C	C00E	108.1(5)
25	H00A	C00C	H00C	108.4
26	H00A	C00C	C00E	110.1
27	H00C	C00C	C00E	110.1
28	Cl01	C00D	C00A	112.2(4)
29	Cl01	C00D	H00D	109.2
30	Cl01	C00D	H00E	109.1
31	C00A	C00D	H00D	109.2
32	C00A	C00D	H00E	109.2
33	H00D	C00D	H00E	107.8
34	C00C	C00E	C00G	119.8(6)
35	C00C	C00E	C00J	119.6(6)
36	C00G	C00E	C00J	120.6(7)
37	O003	C00F	H00F	110.3
38	O003	C00F	H00G	110.3
39	O003	C00F	C00H	106.6(5)
40	H00F	C00F	H00G	108.6
41	H00F	C00F	C00H	110.5

42	H00G	C00F	C00H	110.5
43	C00E	C00G	H00H	120
44	C00E	C00G	C00K	119.9(8)
45	H00H	C00G	C00K	120.1
46	C00F	C00H	H00I	109.4
47	C00F	C00H	H00J	109.5
48	C00F	C00H	H00K	109.5
49	H00I	C00H	H00J	109.5
50	H00I	C00H	H00K	109.5
51	H00J	C00H	H00K	109.5
52	H00L	C00I	C00K	119
53	H00L	C00I	C00L	119
54	C00K	C00I	C00L	122(1)
55	C00E	C00J	H00M	120.2
56	C00E	C00J	C00L	119.8(9)
57	H00M	C00J	C00L	120
58	C00G	C00K	C00I	119(1)
59	C00G	C00K	H00N	121
60	C00I	C00K	H00N	120
61	C00I	C00L	C00J	118(1)
62	C00I	C00L	H00O	121
63	C00J	C00L	H00O	121

Table S6: Torsion angle measurements for Compound 3.

Number	Atom1	Atom2	Atom3	Atom4	Torsion
1	C00C	O002	C009	C006	-2.8(7)
2	C00C	O002	C009	C00B	177.0(5)
3	C009	O002	C00C	H00A	62.3
4	C009	O002	C00C	H00C	-57.2
5	C009	O002	C00C	C00E	-177.4(5)
6	C00F	O003	C008	O004	-2.1(7)
7	C00F	O003	C008	C007	177.5(4)
8	C008	O003	C00F	H00F	54.7
9	C008	O003	C00F	H00G	-65.4
10	C008	O003	C00F	C00H	174.6(5)
11	C00A	N005	C007	C006	0.9(7)
12	C00A	N005	C007	C008	-178.7(4)
13	C007	N005	C00A	C00B	0.3(7)
14	C007	N005	C00A	C00D	-179.2(4)
15	H006	C006	C007	N005	178.9
16	H006	C006	C007	C008	-1.5
17	C009	C006	C007	N005	-1.1(8)
18	C009	C006	C007	C008	178.5(4)
19	H006	C006	C009	O002	0
20	H006	C006	C009	C00B	-179.9
21	C007	C006	C009	O002	180.0(5)

22	C007	C006	C009	C00B	0.1(7)
23	N005	C007	C008	O003	-9.5(6)
24	N005	C007	C008	O004	170.1(5)
25	C006	C007	C008	O003	170.9(4)
26	C006	C007	C008	O004	-9.5(8)
27	O002	C009	C00B	C00A	-179.0(5)
28	O002	C009	C00B	H00B	1
29	C006	C009	C00B	C00A	0.9(8)
30	C006	C009	C00B	H00B	-179.1
31	N005	C00A	C00B	C009	-1.1(8)
32	N005	C00A	C00B	H00B	178.9
33	C00D	C00A	C00B	C009	178.3(5)
34	C00D	C00A	C00B	H00B	-1.7
35	N005	C00A	C00D	Cl01	-86.6(5)
36	N005	C00A	C00D	H00D	152.3
37	N005	C00A	C00D	H00E	34.6
38	C00B	C00A	C00D	Cl01	93.9(5)
39	C00B	C00A	C00D	H00D	-27.2
40	C00B	C00A	C00D	H00E	-144.9
41	O002	C00C	C00E	C00G	64.8(8)
42	O002	C00C	C00E	C00J	-115.7(7)
43	H00A	C00C	C00E	C00G	-174.8
44	H00A	C00C	C00E	C00J	4.6
45	H00C	C00C	C00E	C00G	-55.4
46	H00C	C00C	C00E	C00J	124.1
47	C00C	C00E	C00G	H00H	-1
48	C00C	C00E	C00G	C00K	178.9(8)
49	C00J	C00E	C00G	H00H	179.5
50	C00J	C00E	C00G	C00K	-1(1)
51	C00C	C00E	C00J	H00M	0
52	C00C	C00E	C00J	C00L	179.7(9)
53	C00G	C00E	C00J	H00M	179.2
54	C00G	C00E	C00J	C00L	-1(1)
55	O003	C00F	C00H	H00I	-177.4
56	O003	C00F	C00H	H00J	62.6
57	O003	C00F	C00H	H00K	-57.4
58	H00F	C00F	C00H	H00I	-57.5
59	H00F	C00F	C00H	H00J	-177.5
60	H00F	C00F	C00H	H00K	62.5
61	H00G	C00F	C00H	H00I	62.7
62	H00G	C00F	C00H	H00J	-57.3
63	H00G	C00F	C00H	H00K	-177.3
64	C00E	C00G	C00K	C00I	-1(2)
65	C00E	C00G	C00K	H00N	179
66	H00H	C00G	C00K	C00I	179
67	H00H	C00G	C00K	H00N	-1
68	H00L	C00I	C00K	C00G	-176

69	H00L	C00I	C00K	H00N	4
70	C00L	C00I	C00K	C00G	4(2)
71	C00L	C00I	C00K	H00N	-176
72	H00L	C00I	C00L	C00J	175
73	H00L	C00I	C00L	H00O	-5
74	C00K	C00I	C00L	C00J	-5(2)
75	C00K	C00I	C00L	H00O	175
76	C00E	C00J	C00L	C00I	4(2)
77	C00E	C00J	C00L	H00O	-176
78	H00M	C00J	C00L	C00I	-176
79	H00M	C00J	C00L	H00O	4

Table S7: Bond lengths for Compound 4.

Number	Atom1	Atom2	Cyclicity	Length
1	O001	C00B	acyclic	1.306(3)
2	O001	C00I	acyclic	1.454(4)
3	O002	C00A	acyclic	1.353(3)
4	O002	C00E	acyclic	1.421(3)
5	O003	C00D	acyclic	1.334(4)
6	O003	C00K	acyclic	1.445(4)
7	O004	C00B	acyclic	1.201(4)
8	N005	C007	cyclic	1.340(3)
9	N005	C009	cyclic	1.330(3)
10	O006	C00D	acyclic	1.192(4)
11	C007	C00D	acyclic	1.502(4)
12	C007	C00G	cyclic	1.375(4)
13	C008	C00E	acyclic	1.505(3)
14	C008	C00F	cyclic	1.371(4)
15	C008	C00J	cyclic	1.377(4)
16	C009	C00B	acyclic	1.505(4)
17	C009	C00C	cyclic	1.389(4)
18	C00A	C00C	cyclic	1.382(4)
19	C00A	C00G	cyclic	1.385(4)
20	C00C	H00C	acyclic	0.93
21	C00E	H00A	acyclic	0.97
22	C00E	H00B	acyclic	0.97
23	C00F	H00F	acyclic	0.93
24	C00F	C00H	cyclic	1.387(4)
25	C00G	H00G	acyclic	0.93
26	C00H	H00H	acyclic	0.93
27	C00H	C00L	cyclic	1.367(5)
28	C00I	H00D	acyclic	0.96
29	C00I	H00E	acyclic	0.96
30	C00I	H00I	acyclic	0.96

31	C00J	H00J	acyclic	0.93
32	C00J	C00M	cyclic	1.382(4)
33	C00K	H00K	acyclic	0.96
34	C00K	H00L	acyclic	0.96
35	C00K	H00M	acyclic	0.96
36	C00L	H00N	acyclic	0.93
37	C00L	C00M	cyclic	1.367(5)
38	C00M	H00O	acyclic	0.93

Table S8: Angle measurements for Compound 4.

Number	Atom1	Atom2	Atom3	Angle
1	C00B	O001	C00I	117.2(2)
2	C00A	O002	C00E	117.6(2)
3	C00D	O003	C00K	116.0(2)
4	C007	N005	C009	116.7(2)
5	N005	C007	C00D	114.9(3)
6	N005	C007	C00G	123.5(3)
7	C00D	C007	C00G	121.6(3)
8	C00E	C008	C00F	122.9(3)
9	C00E	C008	C00J	118.7(3)
10	C00F	C008	C00J	118.4(3)
11	N005	C009	C00B	117.6(3)
12	N005	C009	C00C	124.1(3)
13	C00B	C009	C00C	118.2(3)
14	O002	C00A	C00C	125.3(3)
15	O002	C00A	C00G	116.2(3)
16	C00C	C00A	C00G	118.6(3)
17	O001	C00B	O004	124.6(3)
18	O001	C00B	C009	112.4(3)
19	O004	C00B	C009	122.9(3)
20	C009	C00C	C00A	118.1(3)
21	C009	C00C	H00C	121
22	C00A	C00C	H00C	121
23	O003	C00D	O006	124.3(3)
24	O003	C00D	C007	111.8(3)
25	O006	C00D	C007	123.9(3)
26	O002	C00E	C008	109.1(2)
27	O002	C00E	H00A	109.9
28	O002	C00E	H00B	109.9
29	C008	C00E	H00A	109.9
30	C008	C00E	H00B	109.9
31	H00A	C00E	H00B	108.3
32	C008	C00F	H00F	119.7
33	C008	C00F	C00H	120.7(3)

34	H00F	C00F	C00H	119.7
35	C007	C00G	C00A	119.0(3)
36	C007	C00G	H00G	120.5
37	C00A	C00G	H00G	120.5
38	C00F	C00H	H00H	119.8
39	C00F	C00H	C00L	120.4(3)
40	H00H	C00H	C00L	119.8
41	O001	C00I	H00D	109.4
42	O001	C00I	H00E	109.5
43	O001	C00I	H00I	109.5
44	H00D	C00I	H00E	109.4
45	H00D	C00I	H00I	109.5
46	H00E	C00I	H00I	109.5
47	C008	C00J	H00J	119.6
48	C008	C00J	C00M	120.8(3)
49	H00J	C00J	C00M	119.6
50	O003	C00K	H00K	109.5
51	O003	C00K	H00L	109.5
52	O003	C00K	H00M	109.5
53	H00K	C00K	H00L	109.5
54	H00K	C00K	H00M	109.5
55	H00L	C00K	H00M	109.5
56	C00H	C00L	H00N	120.4
57	C00H	C00L	C00M	119.3(3)
58	H00N	C00L	C00M	120.4
59	C00J	C00M	C00L	120.4(3)
60	C00J	C00M	H00O	119.8
61	C00L	C00M	H00O	119.8

Table S9: Torsion angle measurements for Compound 4.

Number	Atom1	Atom2	Atom3	Atom4	Torsion
1	C00I	O001	C00B	O004	1.1(5)
2	C00I	O001	C00B	C009	-179.3(2)
3	C00B	O001	C00I	H00D	-63.6
4	C00B	O001	C00I	H00E	176.4
5	C00B	O001	C00I	H00I	56.4
6	C00E	O002	C00A	C00C	4.1(4)
7	C00E	O002	C00A	C00G	-175.8(3)
8	C00A	O002	C00E	C008	178.8(2)
9	C00A	O002	C00E	H00A	58.3
10	C00A	O002	C00E	H00B	-60.7
11	C00K	O003	C00D	O006	0.7(5)
12	C00K	O003	C00D	C007	-179.8(2)
13	C00D	O003	C00K	H00K	-169

14	C00D	O003	C00K	H00L	70.9
15	C00D	O003	C00K	H00M	-49
16	C009	N005	C007	C00D	179.4(3)
17	C009	N005	C007	C00G	0.1(4)
18	C007	N005	C009	C00B	179.5(3)
19	C007	N005	C009	C00C	-1.1(4)
20	N005	C007	C00D	O003	172.8(3)
21	N005	C007	C00D	O006	-7.7(5)
22	C00G	C007	C00D	O003	-7.9(4)
23	C00G	C007	C00D	O006	171.6(3)
24	N005	C007	C00G	C00A	1.0(5)
25	N005	C007	C00G	H00G	-179
26	C00D	C007	C00G	C00A	-178.2(3)
27	C00D	C007	C00G	H00G	1.7
28	C00F	C008	C00E	O002	6.2(4)
29	C00F	C008	C00E	H00A	126.7
30	C00F	C008	C00E	H00B	-114.3
31	C00J	C008	C00E	O002	-175.1(3)
32	C00J	C008	C00E	H00A	-54.6
33	C00J	C008	C00E	H00B	64.4
34	C00E	C008	C00F	H00F	-0.6
35	C00E	C008	C00F	C00H	179.4(3)
36	C00J	C008	C00F	H00F	-179.3
37	C00J	C008	C00F	C00H	0.7(5)
38	C00E	C008	C00J	H00J	0.8
39	C00E	C008	C00J	C00M	-179.2(3)
40	C00F	C008	C00J	H00J	179.6
41	C00F	C008	C00J	C00M	-0.5(5)
42	N005	C009	C00B	O001	8.4(4)
43	N005	C009	C00B	O004	-172.0(3)
44	C00C	C009	C00B	O001	-171.1(3)
45	C00C	C009	C00B	O004	8.5(5)
46	N005	C009	C00C	C00A	0.8(4)
47	N005	C009	C00C	H00C	-179.2
48	C00B	C009	C00C	C00A	-179.7(3)
49	C00B	C009	C00C	H00C	0.2
50	O002	C00A	C00C	C009	-179.5(3)
51	O002	C00A	C00C	H00C	0.6
52	C00G	C00A	C00C	C009	0.4(4)
53	C00G	C00A	C00C	H00C	-179.6
54	O002	C00A	C00G	C007	178.6(3)
55	O002	C00A	C00G	H00G	-1.3
56	C00C	C00A	C00G	C007	-1.2(5)
57	C00C	C00A	C00G	H00G	178.8
58	C008	C00F	C00H	H00H	179.7
59	C008	C00F	C00H	C00L	-0.4(5)
60	H00F	C00F	C00H	H00H	-0.3

61	H00F	C00F	C00H	C00L	179.7
62	C00F	C00H	C00L	H00N	179.7
63	C00F	C00H	C00L	C00M	-0.3(5)
64	H00H	C00H	C00L	H00N	-0.3
65	H00H	C00H	C00L	C00M	179.7
66	C008	C00J	C00M	C00L	-0.1(5)
67	C008	C00J	C00M	H00O	179.8
68	H00J	C00J	C00M	C00L	179.8
69	H00J	C00J	C00M	H00O	-0.2
70	C00H	C00L	C00M	C00J	0.5(6)
71	C00H	C00L	C00M	H00O	-179.5
72	H00N	C00L	C00M	C00J	-179.5
73	H00N	C00L	C00M	H00O	0.6

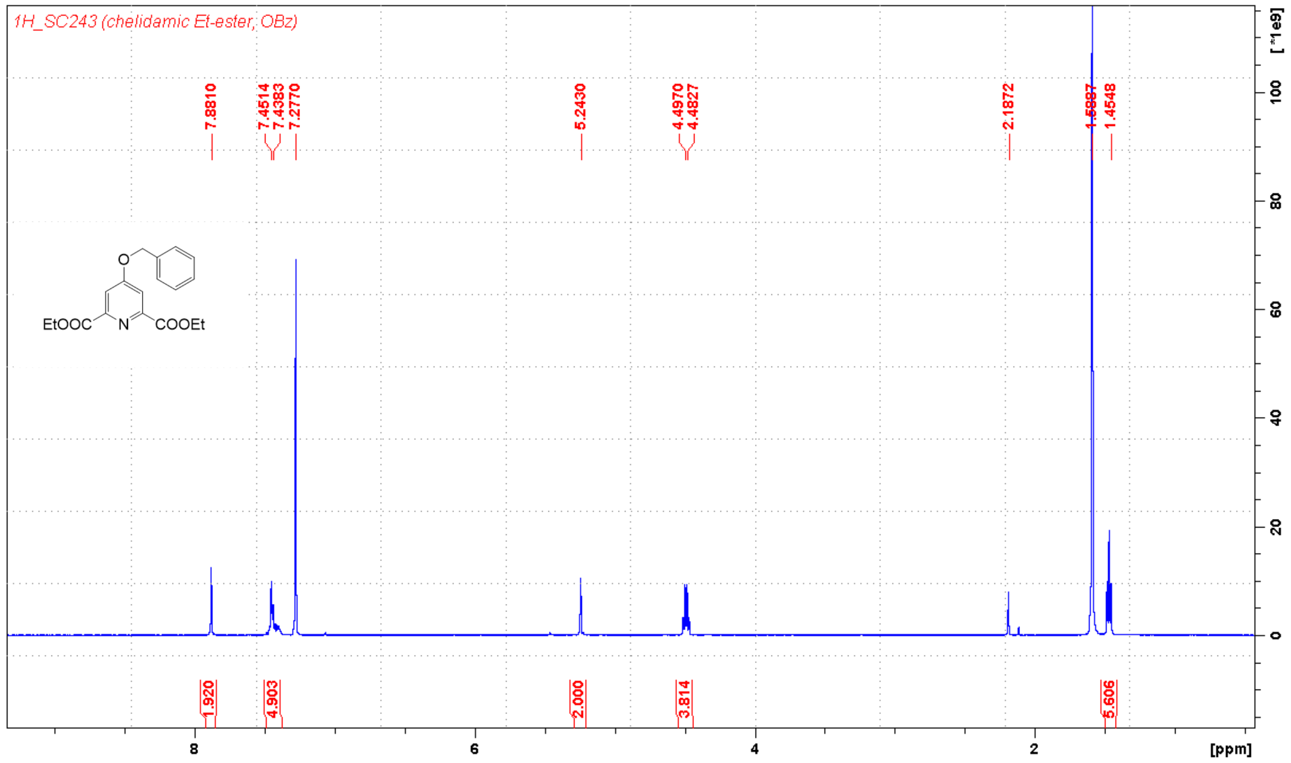

Figure S1: ^{1}H NMR spectrum of Compound 1.

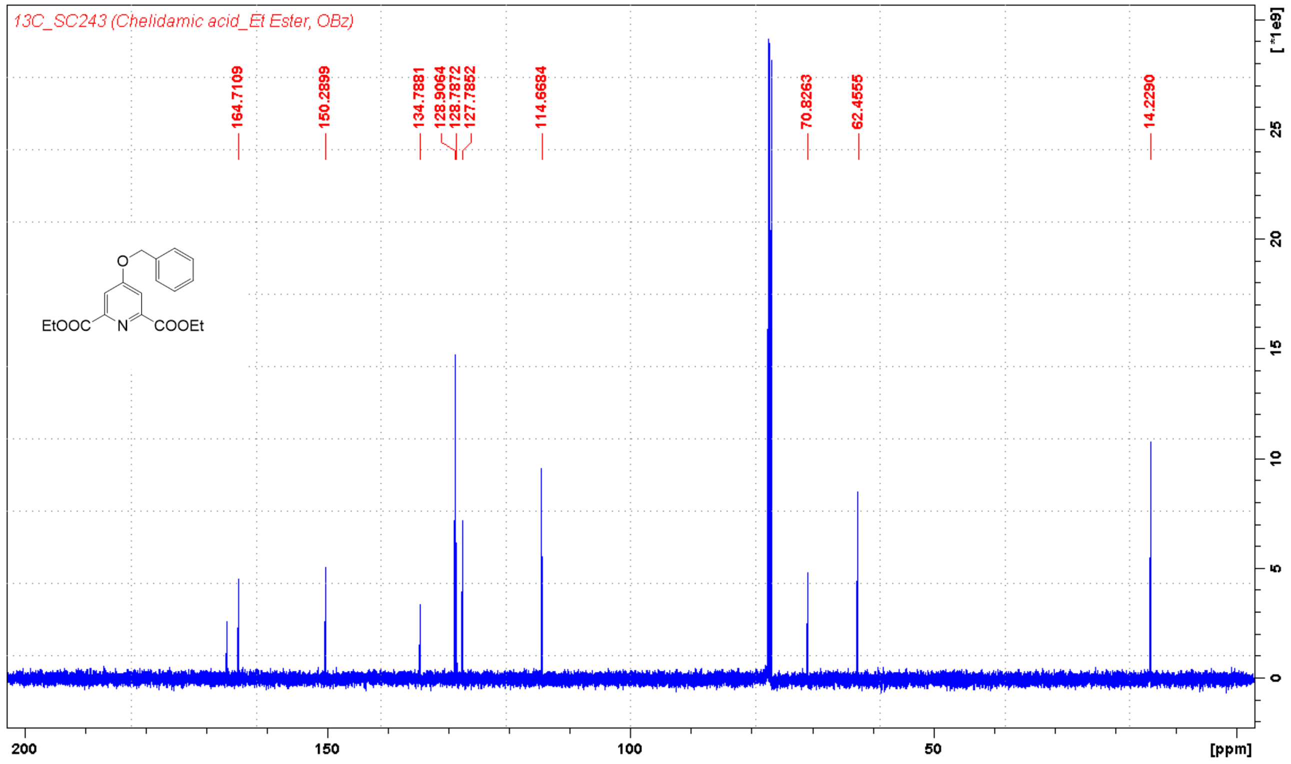

Figure S2: ^{13}C NMR spectrum of Compound 1.

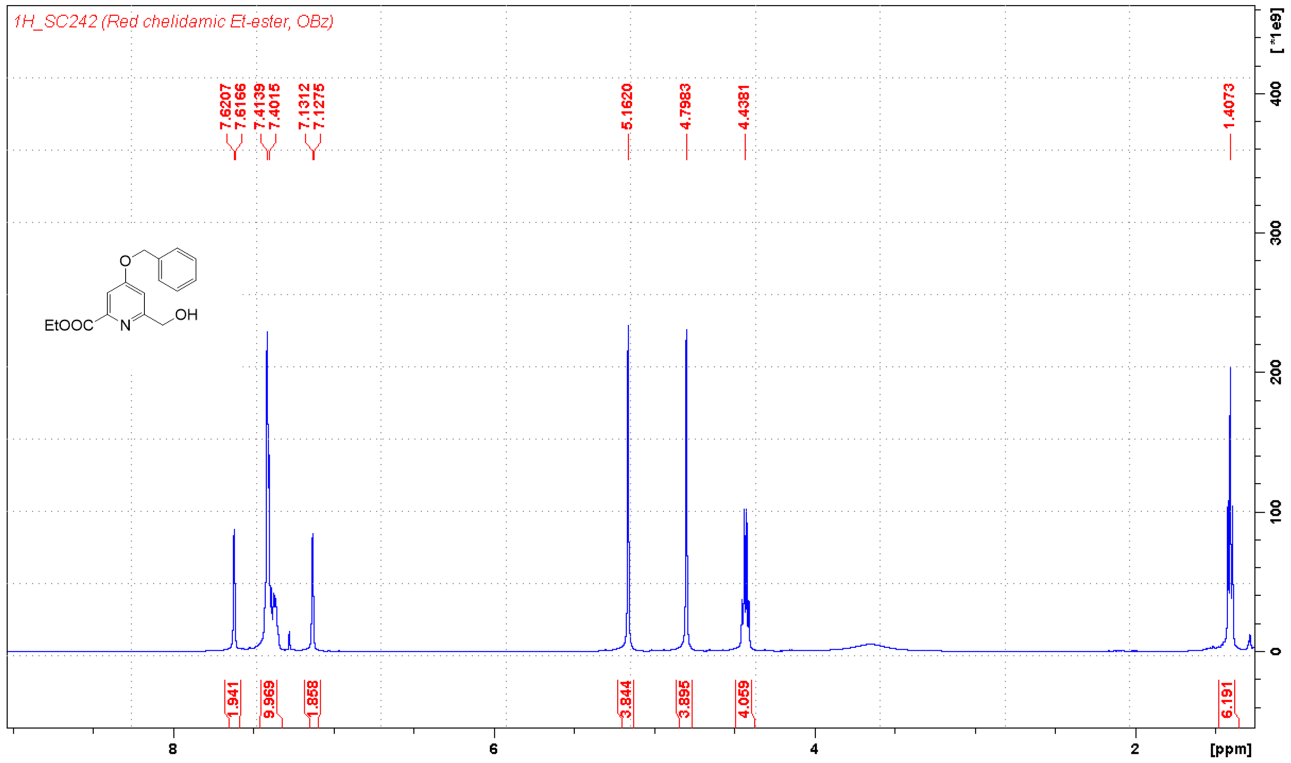

Figure S3: ^{1}H NMR spectrum of Compound 2.

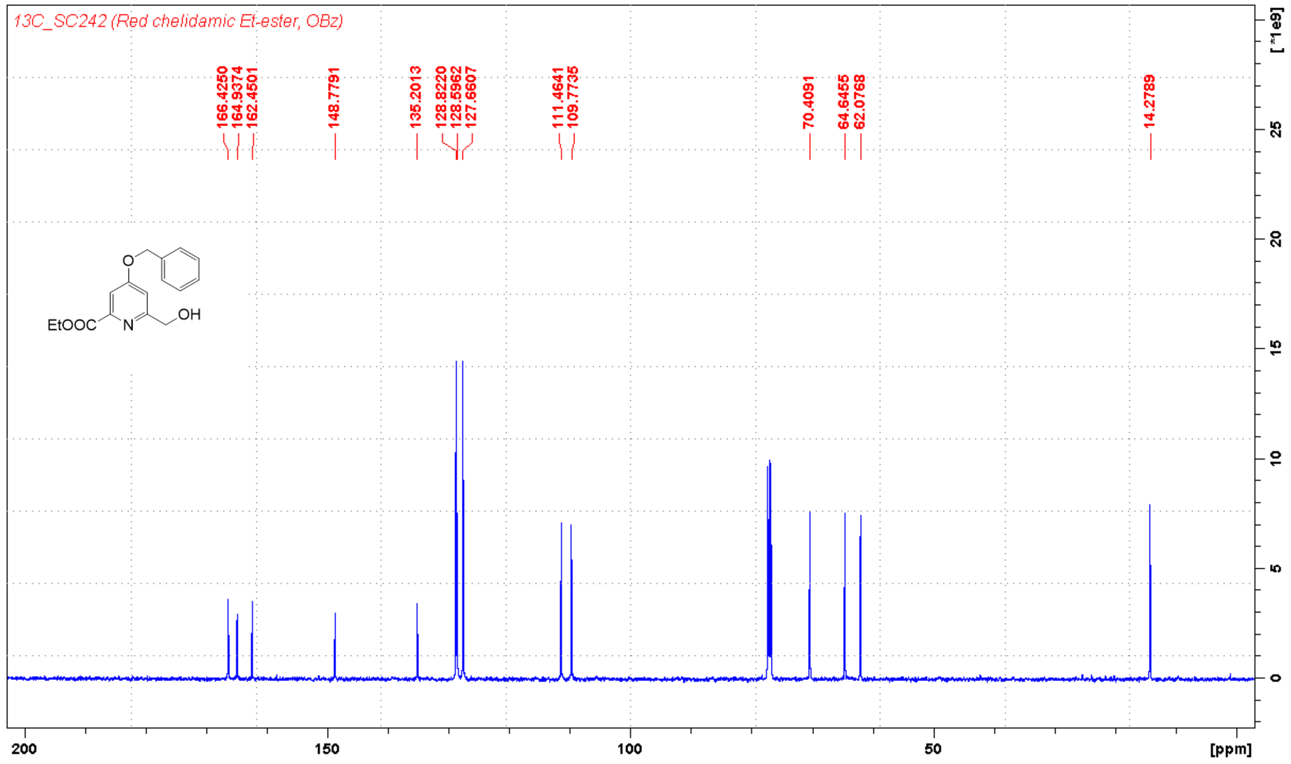

Figure S4: ^{13}C NMR spectrum of Compound 2.

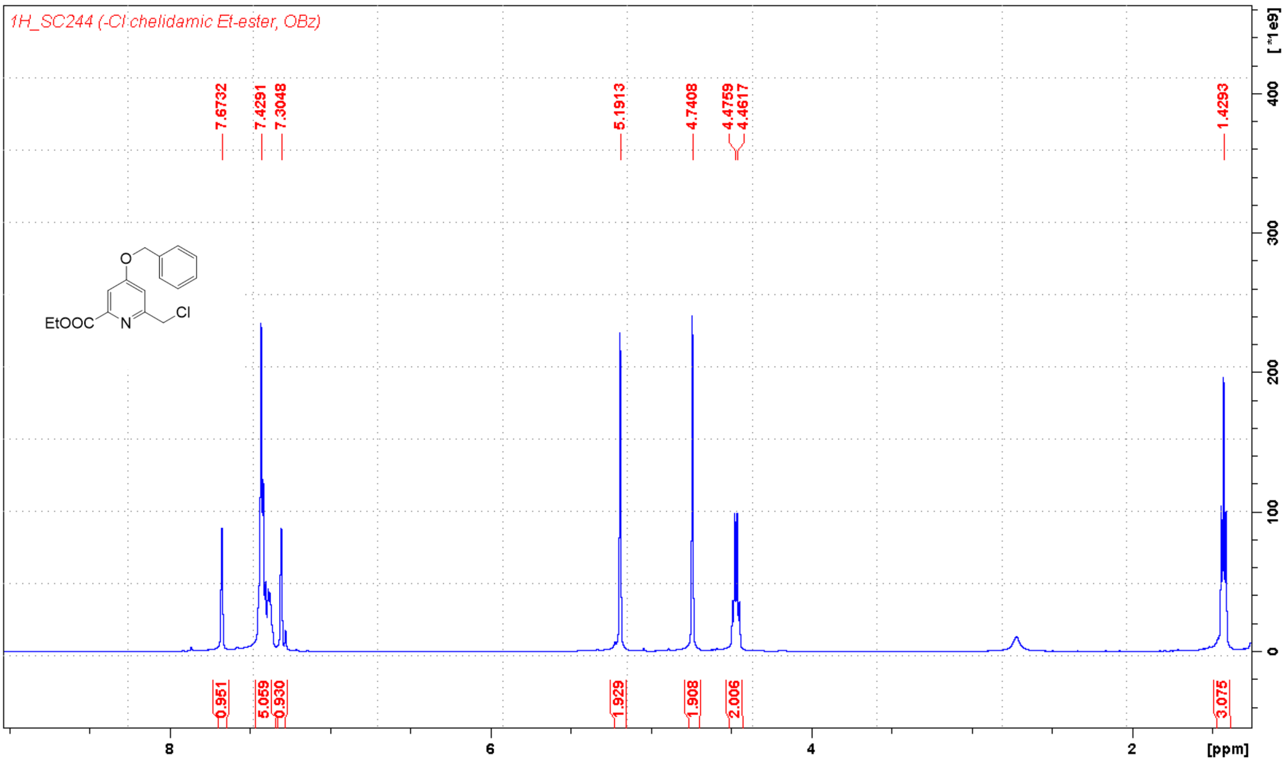

Figure S5: ^{1}H NMR spectrum of Compound 3.

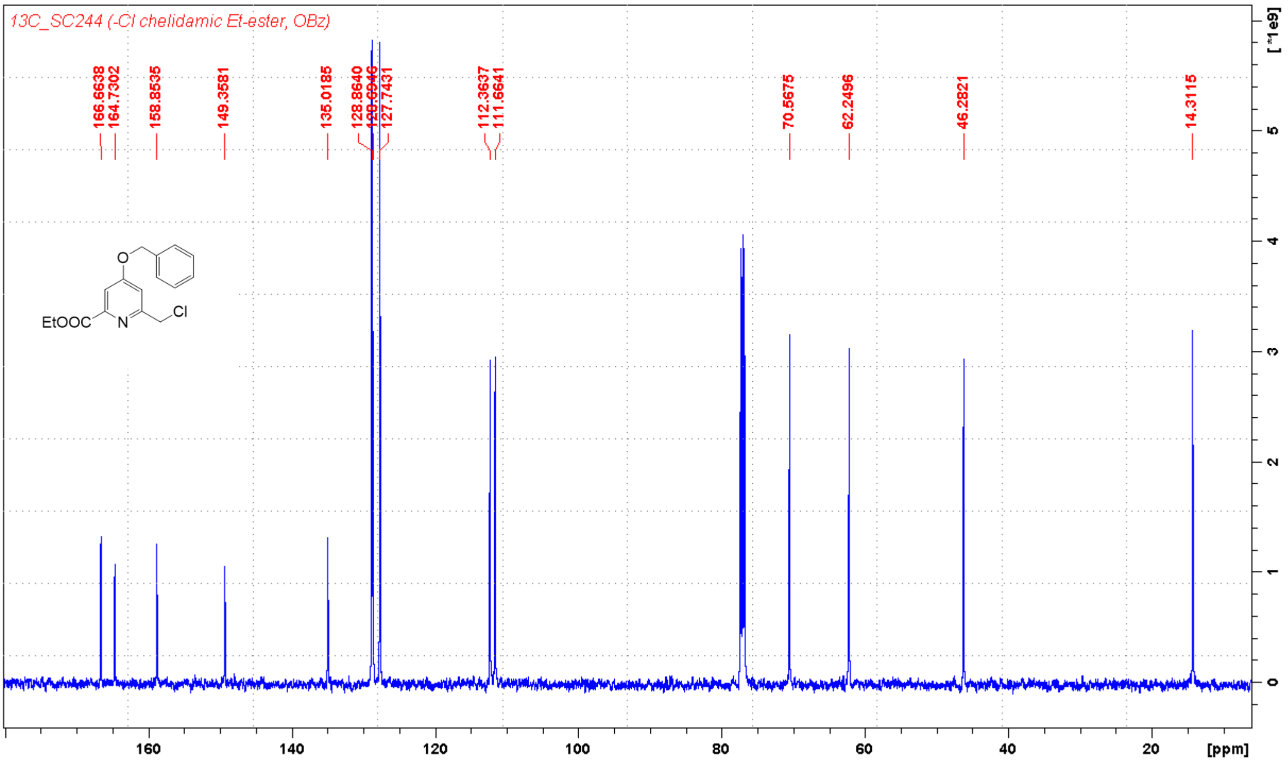

Figure S6: ^{13}C NMR spectrum of Compound 3.

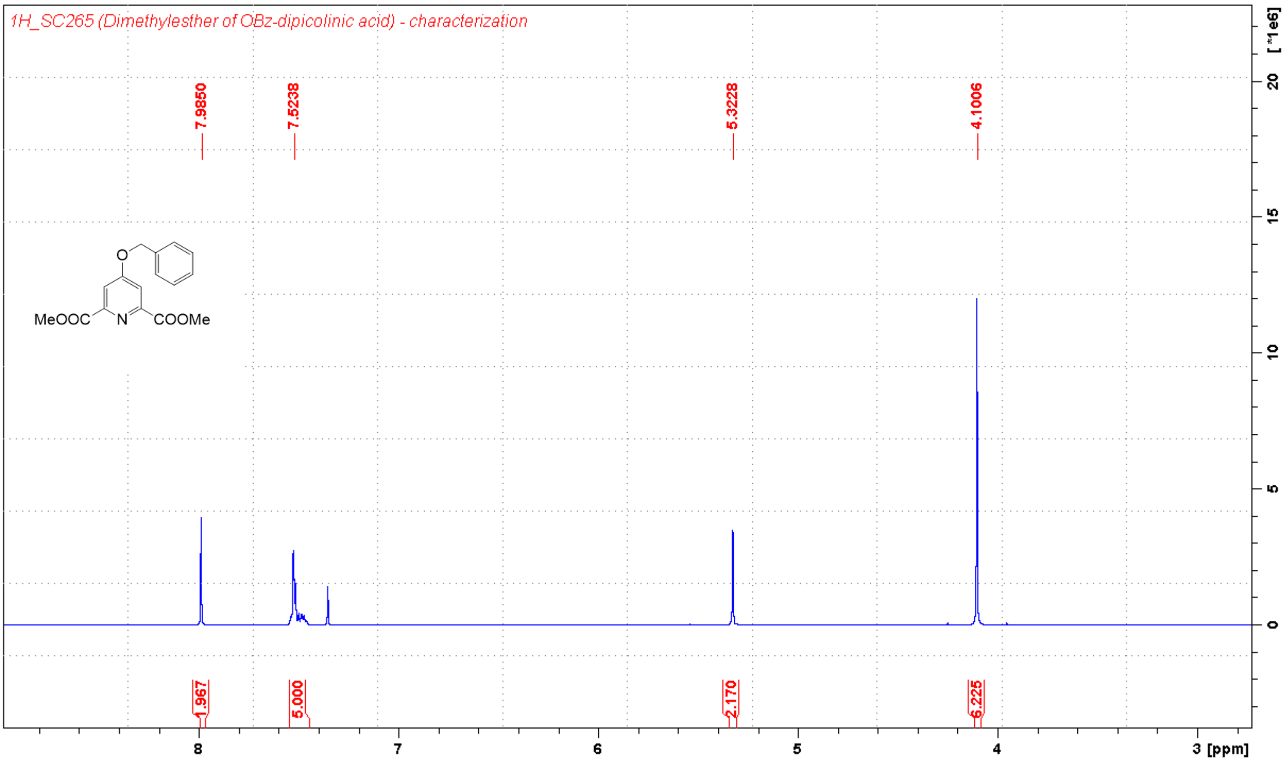

Figure S7: ^{1}H NMR spectrum of Compound 4.

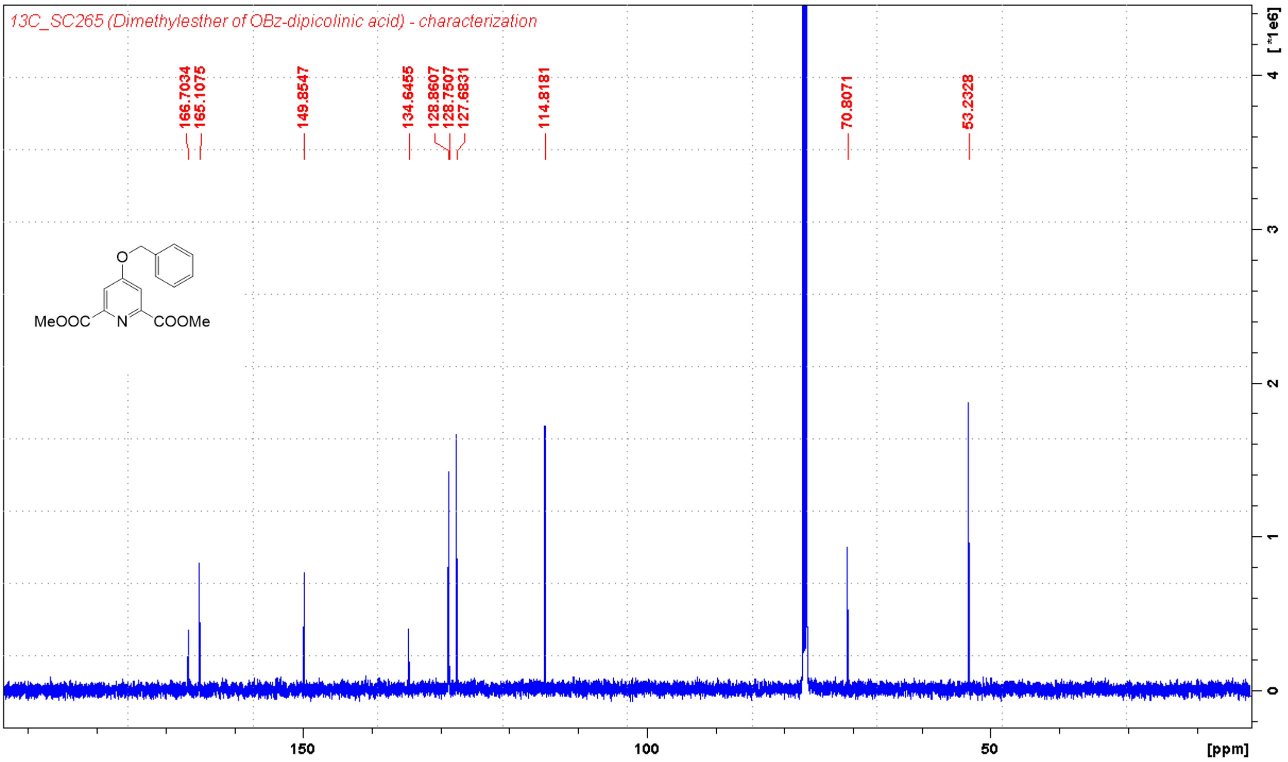

Figure S8: ^{13}C NMR spectrum of Compound 4.

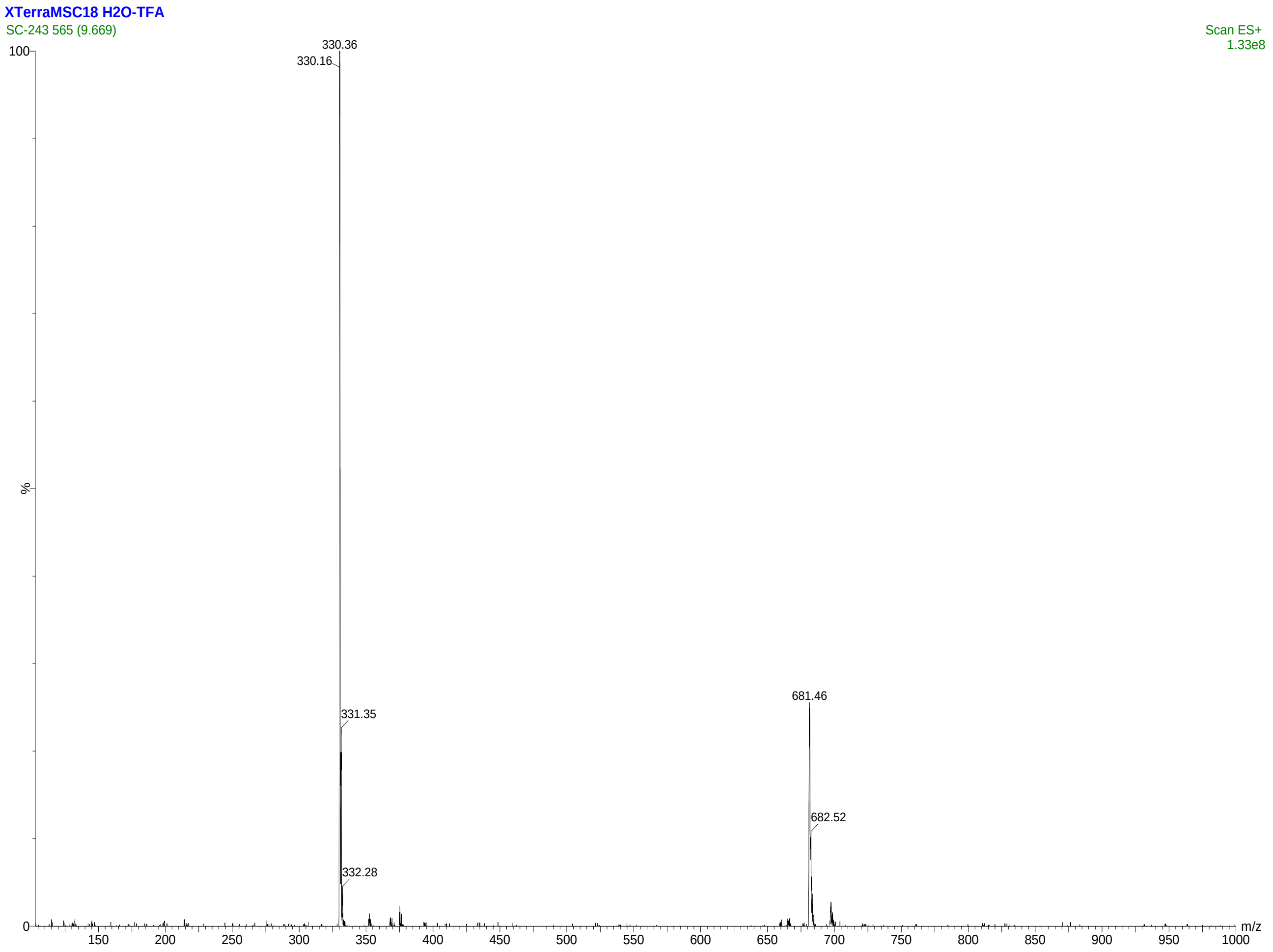

Figure S9: Mass spectrum of Compound 1, displaying the molecular ion peak ($M+H^+$) and the dimeric adduct ($2M+Na^+$).

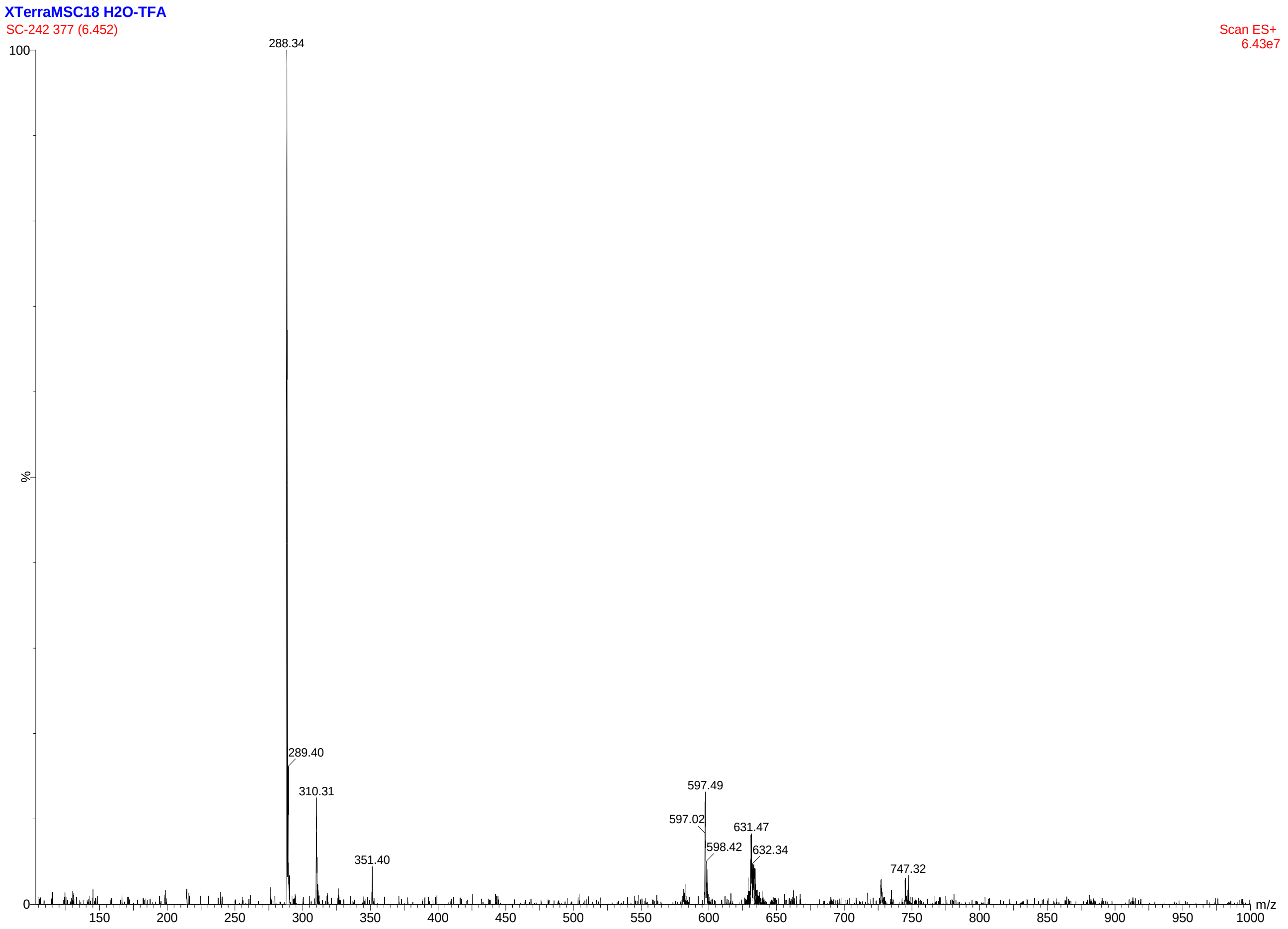

Figure S10: Mass spectrum of Compound 2, displaying the molecular ion peak ($M+H^+$) and the dimeric adduct ($2M+Na^+$).

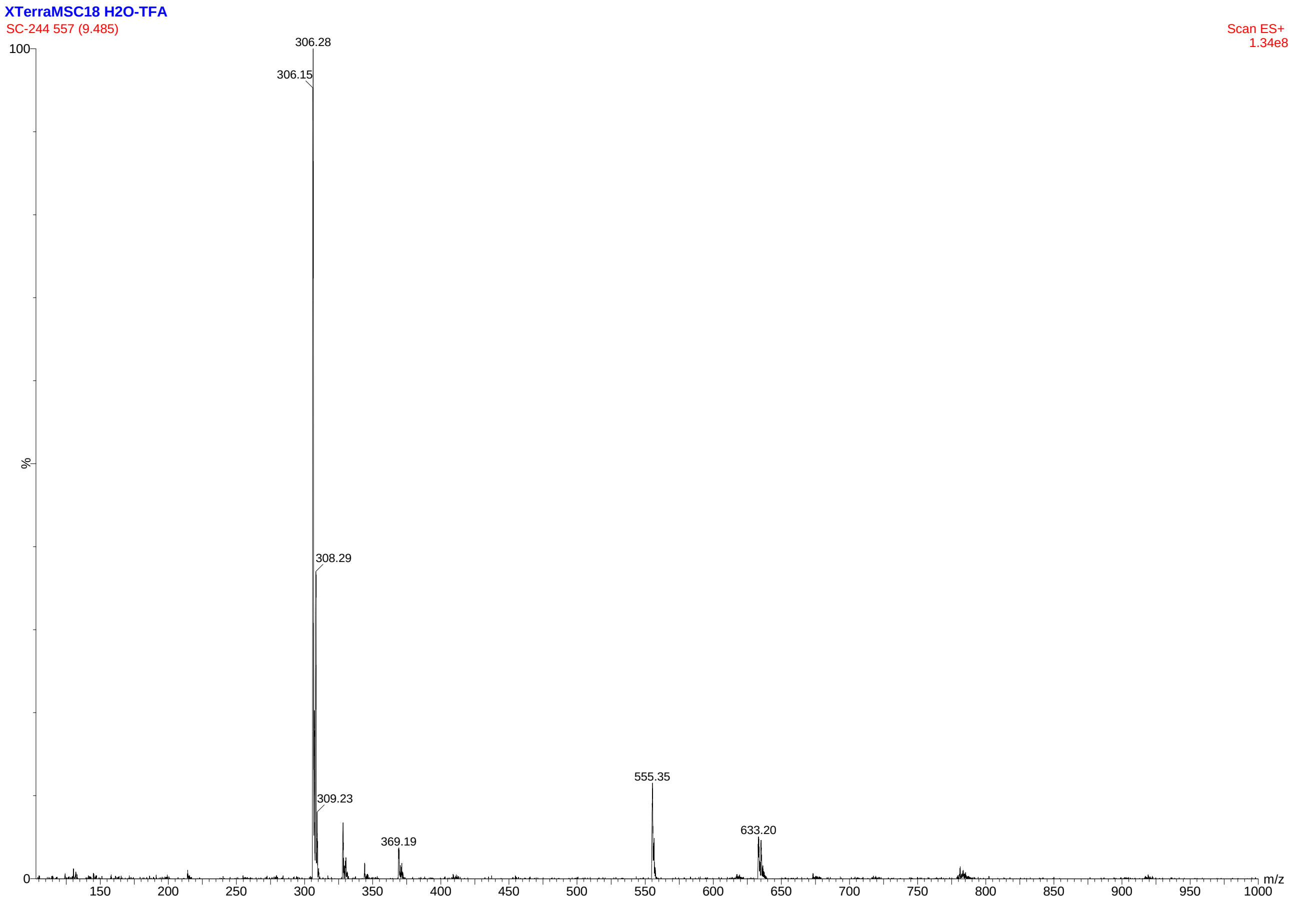

Figure S11: Mass spectrum of Compound 3, displaying the molecular ion peak ($M+H^+$) and the dimeric adduct ($2M+Na^+$).

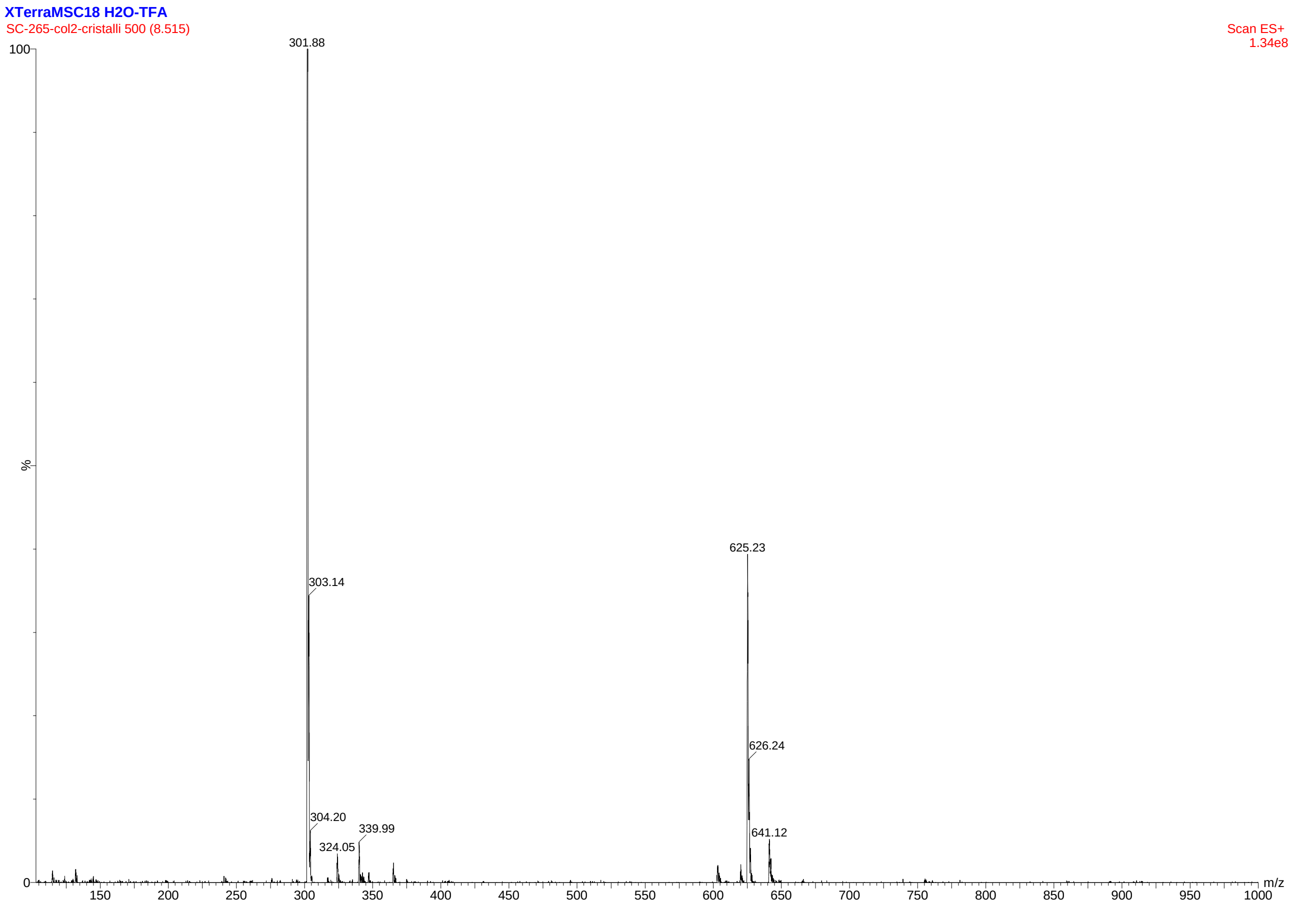

Figure S11: Mass spectrum of Compound 4, displaying the molecular ion peak (M+H$^+$) and the dimeric adduct (2M+H$^+$).

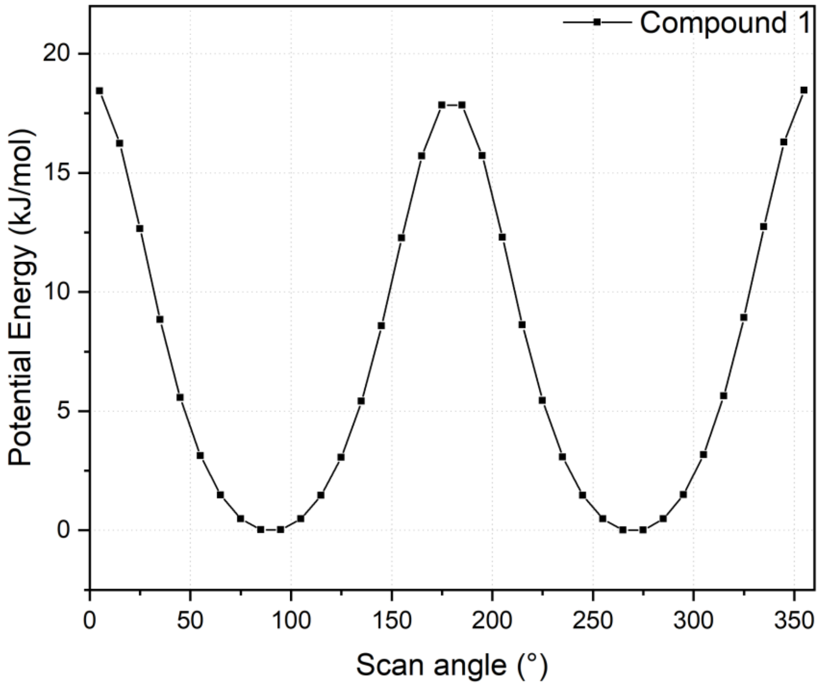

Figure S12: Potential Energy Scan of Compound 1.

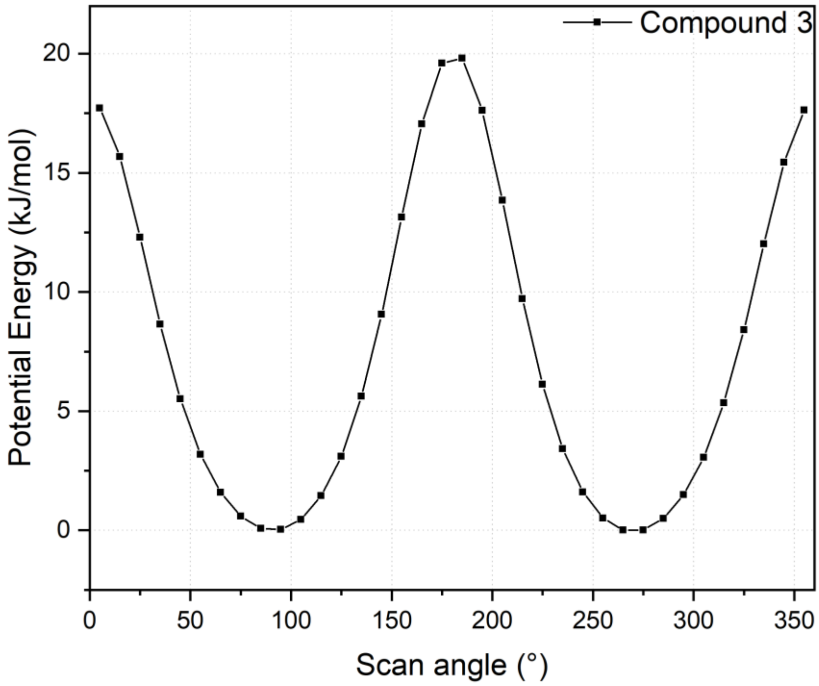

Figure S13: Potential Energy Scan of Compound 3.

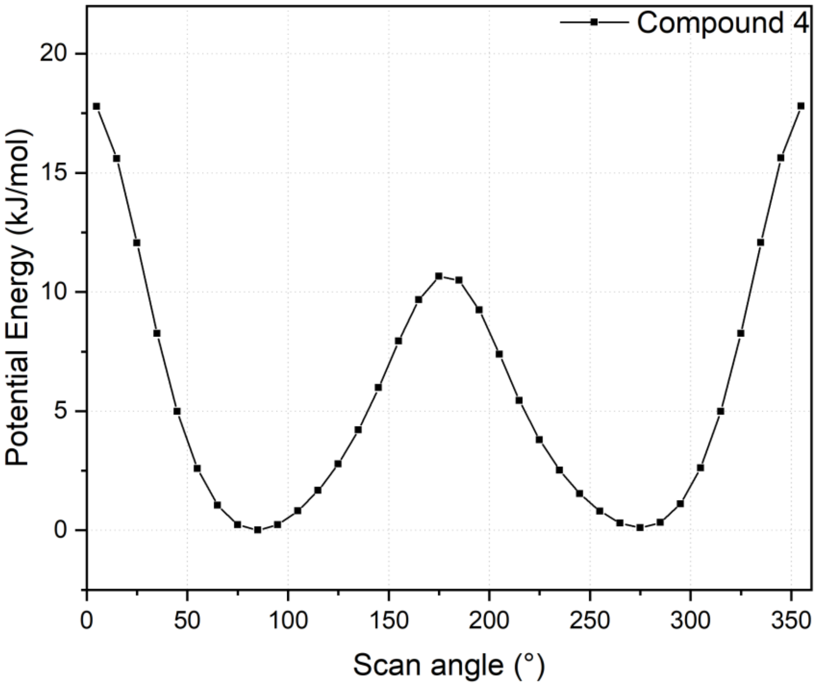

Figure S14: Potential Energy Scan of Compound 4.
